# 8-Oxoguanine targeted by 8-oxoguanine DNA glycosylase 1 (OGG1) is central to fibrogenic gene activation upon lung injury

**DOI:** 10.1093/nar/gkac1241

**Published:** 2023-01-18

**Authors:** Lang Pan, Wenjing Hao, Yaoyao Xue, Ke Wang, Xu Zheng, Jixian Luo, Xueqing Ba, Yang Xiang, Xiaoqun Qin, Jesper Bergwik, Lloyd Tanner, Arne Egesten, Allan R Brasier, Istvan Boldogh

**Affiliations:** Department of Microbiology and Immunology, University of Texas Medical Branch, Galveston, TX 77555, USA; Department of Microbiology and Immunology, University of Texas Medical Branch, Galveston, TX 77555, USA; Institute of Genetics and Developmental Biology, Chinese Academy of Sciences, Beijing 100871, China; Department of Microbiology and Immunology, University of Texas Medical Branch, Galveston, TX 77555, USA; Department of Microbiology and Immunology, University of Texas Medical Branch, Galveston, TX 77555, USA; Department of Microbiology and Immunology, University of Texas Medical Branch, Galveston, TX 77555, USA; Department of Microbiology and Immunology, University of Texas Medical Branch, Galveston, TX 77555, USA; School of Life Sciences, Shanxi University, Taiyuan, Shanxi 030006, China; Department of Microbiology and Immunology, University of Texas Medical Branch, Galveston, TX 77555, USA; Key Laboratory of Molecular Epigenetics of Ministry of Education, School of Life Science, Northeast Normal University, Changchun, Jilin 130024, China; Department of Physiology, School of Basic Medical Science, Central South University, Changsha, Hunan 410000, China; Department of Physiology, School of Basic Medical Science, Central South University, Changsha, Hunan 410000, China; Respiratory Medicine, Allergology, & Palliative Medicine, Department of Clinical Sciences Lund, Lund University and Skåne University Hospital, SE-221 84 Lund, Sweden; Respiratory Medicine, Allergology, & Palliative Medicine, Department of Clinical Sciences Lund, Lund University and Skåne University Hospital, SE-221 84 Lund, Sweden; Respiratory Medicine, Allergology, & Palliative Medicine, Department of Clinical Sciences Lund, Lund University and Skåne University Hospital, SE-221 84 Lund, Sweden; Department of Medicine, University of Wisconsin-Madison School of Medicine and Public Health (SMPH), Madison, WI 53705, USA; Department of Microbiology and Immunology, University of Texas Medical Branch, Galveston, TX 77555, USA

## Abstract

Reactive oxygen species (ROS) are implicated in epithelial cell-state transition and deposition of extracellular matrix upon airway injury. Of the many cellular targets of ROS, oxidative DNA modification is a major driving signal. However, the role of oxidative DNA damage in modulation profibrotic processes has not been fully delineated. Herein, we report that oxidative DNA base lesions, 8-oxoG, complexed with 8-oxoguanine DNA glycosylase 1 (OGG1) functions as a pioneer factor, contributing to transcriptional reprogramming within airway epithelial cells. We show that TGFβ1-induced ROS increased 8-oxoG levels in open chromatin, dynamically reconfigure the chromatin state. OGG1 complexed with 8-oxoG recruits transcription factors, including phosphorylated SMAD3, to pro-fibrotic gene promoters thereby facilitating gene activation. Moreover, 8-oxoG levels are elevated in lungs of mice subjected to TGFβ1-induced injury. Pharmacologic targeting of OGG1 with the selective small molecule inhibitor of 8-oxoG binding, TH5487, abrogates fibrotic gene expression and remodeling in this model. Collectively, our study implicates that 8-oxoG substrate-specific binding by OGG1 is a central modulator of transcriptional regulation in response to tissue repair.

## INTRODUCTION

Following injury, the airway epithelium undergoes a spectrum of phenotypic changes through a process called epithelial-mesenchymal transition (EMT) (1). In contrast to the events occurring during embryogenesis and metastasis, this type of cell-state transition is associated with microinjury-maintained inflammation in terminally differentiated epithelial surfaces, referred to as type II EMT (2,3). During acute and unresolved inflammation, hyperactivity of oxidoreductases generates reactive oxygen species (ROS) that are essential participants in intracellular regulation (4). Additionally, growth factors and cytokines produced during injury/repair also promote ROS generation (5,6). Over-production of ROS and reactive nitrogen species (RNS) leads to oxidative and nitrosative stress. Nuclear DNA oxidation is one of the sensors transducing signals from oxidants into biological responses (7). However, comprehensive investigations into inherent changes in DNA oxidation during type II EMT in a non-neoplastic setting, free of transformation-associated oncogenes are still lacking.

In DNA, ROS cause base-specific modifications in GC-rich sequences. Guanine has the lowest oxidation potential and its oxidation primarily yields 7,8-dihydro-8-oxoguanine (8-oxoG), which is a biomarker of oxidative DNA damage (8). 8-oxoG is specifically excised by 8-oxoguanine DNA glycosylase (OGG1), responsible for first step in activating the base excision repair (BER) pathway to maintain genome fidelity (9). It has been well established that 8-oxoG selectively accumulates within regulatory regions under oxidative stress, such as promoters and super-enhancers (10–12), which ascribes an epigenetic-like role to 8-oxoG other than as a mutagen in cellular oxidation. Interestingly, Ogg1 null mice (*Ogg1*^−/−^) accumulate abnormal levels of 8-oxoG in the genome, but exhibit altered responses to environmental stimuli (13–18). These findings suggest not only 8-oxoG itself, but also additional recognition by its repair protein OGG1 is responsible for activating selective transcriptional programs. Our group and others have reported that 8-oxoG at gene promoters, in conjunction with BER processes initiated by OGG1, regulates transcription (19–24). The first evidence implicating OGG1 in fibrotic gene activation came from whole transcriptome analysis, where we found that OGG1-BER-driven signaling triggers airway remodeling programs (25,26). Administration of the OGG1 inhibitor, TH5487, which displaces 8-oxoG from the active binding site of OGG1, decreased tissue remodeling and bleomycin-induced pulmonary fibrosis in murine models (27,28). However, the mechanism of transcriptional reprogramming orchestrated by 8-oxoG-OGG1 complex in fibrotic gene activation remains poorly understood.

Herein, we have established a model of type II EMT, whereby OGG1 initiates fibrotic gene transcription through its ability to bind DNA substrates in close proximity to *cis*-acting element(s) in promoters. Experiments employing genetic loss of function and pharmacological manipulation revealed a direct transcriptional control of type II EMT by OGG1. Additionally, we show that SMAD-DNA binding is enhanced by the OGG1-DNA complex, providing a compelling explanation for how oxidative stress is an essential intracellular signal utilized in transforming growth factor β1 (TGFβ1)-dependent transcriptional activation. Furthermore, functional inhibition of OGG1 with TH5487 markedly attenuates fibrotic responses in surrounding airways in a murine model of lung injury. Overall, these data demonstrate an unappreciated role of DNA repair proteins’ contribution to epigenetic regulation and provide proof of concept for therapeutic targeting in fibrosing disorders.

## MATERIALS AND METHODS

### Cell culture and inhibitors treatment

Immortalized human small airway epithelial cells (hSAECs) were established by expressing human telomerase reverse transcriptase (hTERT) and cyclin-dependent kinase-4 retrovirus constructs (29). Cells were maintained in serum-free growth medium that contained supplements developed for the cultivation of primary epithelial cells from the distal respiratory system. The hSAECs in our study are not a transformed cell line and should be relatively free of epigenetic changes that might arise secondary to neoplastic transformation. Human diploid fibroblast (MRC5) cells from ATCC were maintained in Minimum Essential Medium. *Ogg1*^+/+^ and *Ogg1*^−/−^ mouse embryo fibroblast (MEF) cells (13) are maintained in Dulbecco's Modified Eagle's Medium, supplemented with 10% fetal bovine serum, penicillin, and streptomycin. All cultures were negative for mycoplasma contamination.

EMT was induced in hSAECs by consecutive challenges of 10 ng/ml of TGFβ1 as described in previous publication (30). Cells were harvested after exposure for one hour. Where necessary, cells were pretreated with *N*-*tert*-butyl-α-phenylnitrone (PBN, 100 μM), TH5487 (10 μM), SU0268 (1 μM), TH2840 (10 μM), JQ1 (0.5 μM), O8 (10 μM), BMS345541 (5 μM), OG-L002 (0.5 μM), Apurinic Endonuclease 1 Inhibitor III (APE inhibitor III, 1 μM) and E3330 (10 μM) for 1 h. In long-term treatment, TH5487 and SU0268 were added daily in fresh medium. OGG1 inhibition by TH5487 and SU0268 persists over 24 h (20,31).

CRISPR (clustered regularly interspaced short palindromic repeats)/Cas9 knockout (KO) of OGG1 was generated essentially as previously described (20). Briefly, two OGG1 targeting sequences 5′-GATGCGGGCGATGTTGTTGTTGG-3′ and 5′-AACAACATCGCCCGCATCACTGG-3′ were cloned into pSpCas9(BB)-2A-Puro plasmid. hSAECs stably expressed empty vector and OGG1-KO plasmid were selected and maintained with 2 μg/ml of puromycin in small airway epithelial cell growth medium. Detailed information of the reagents used in this study are listed in Supplementary Tables S1.

### ROS measurements

ROS levels were determined by the ROS sensitive fluorogenic probe Amplex Red and 2',7'-dihydro-dichlorofluorescein diacetate (H_2_DCF-DA) according to a previous protocol (32). In brief, hSAECs were grown to 70% confluence in 24-well plates at time of TGFβ1 addition. At 3 min before the end of the incubation time at 37°C, Amplex Red was added to a final concentration of 100 μM. H_2_DCF-DA was added to a final concentration of 5 μM. Cells were lysed in 500 μl of RIPA buffer and analyzed immediately using BioTek Synergy H1 reader. Fluorescence was measured with excitation of 540 nm and emission of 590 nm for Amplex Red assay, excitation of 488 nm and emission of 510 nm for H_2_DCF-DA assay.

### Immunofluorescence (IF) and proximity ligation assay (PLA)

Cells growing on microscope coverslips were washed with PBS, air dried, and fixed in (1:1) acetone-methanol. After being rehydrated in PBS for 15 min, the cells were blocked with 1% BSA for 60 min at room temperature (RT). Following incubation with indicated primary antibody (Ab) at 4°C overnight, the cells were washed three times with PBS for 15 min and then exposed to fluorescein-conjugated secondary Ab for 60 min at RT in the dark. For isotype control, the primary Ab was omitted. After being triple washed with PBS, the DNA was stained with DAPI (10 ng/ml) for 15 min. The coverslips were finally mounted in antifade medium on a microscope slide for microscopy (OLYMPUS Microscope System (BX53P) with a built-in digital CCD color camera DP73WDR).

For visualization of 8-oxoG in nuclei, immunocytochemistry was performed according to a previously described protocol (33). Briefly, fixed cells were treated with 100 μg/ml RNase A in TEN buffer (10 mM Tris–HCl, pH 7.4, and 1 mM ethylenediaminetetraacetic acid (EDTA), pH 7.6, 400 mM NaCl) for 60 min at 37°C to eliminate the binding of antibody to RNA containing 8-oxoG. Coverslips were then washed one time in PBS and incubated with 10 mg/ml proteinase K in 100 mM Tris–HCl, pH 8.0, and 50 mM EDTA, pH 7.6, for 10 min at RT. To provide maximal exposure of Ab to regions of DNA where 8-oxoG lesions may exist, DNA was denatured at RT with 1.5 N HCl for 15 min and then neutralized with 2.5 volumes of 1 M Tris-base for an additional 7 min before proceeding to the IF protocol, as described above.

PLA was performed with Duolink^®^*In Situ* PLA^®^ kit according to the protocol online (https://www.sigmaaldrich.com/US/en/technical-documents/protocol/protein-biology/protein-and-nucleic-acid-interactions/duolink-fluorescence-user-manual). Briefly, Flag-OGG1 (34) (0.5 μg/35-cm petri dish) expressing hSAECs were mock and TGFβ1 treated. Anti-Flag (mouse origin, 1:100) and -pSMAD3 (Rabbit origin, 1:100) were used as primary antibodies and incubated at 4°C overnight. PLUS and MINUS PLA probes were incubated at 37°C for 1 h, followed by ligation and Polymerase amplification at 37°C for 2 h. Between each step, samples were washed extensively according to instructions. Total nuclei and PLA spots were quantified with ImageJ, and nuclear or cytoplasmic PLA speckles were quantified according to the protocol online (https://microscopy.duke.edu/guides/count-nuclear-foci-ImageJ).

### Quantitative real-time PCR (qRT-PCR) and PCR arrays

Total RNA was extracted using RNeasy extraction kit. Complementary DNAs (cDNAs) were generated using the one-step iScript cDNA Synthesis Kit. qRT-PCR was performed with cDNA samples using iTaq Universal SYBR green Supermix and the signal was detected using CFX96 real-time PCR system (Bio-Rad, CA, USA). Expression analysis of EMT pathway was analyzed by plate-based quantitative PCR arrays (Mouse Epithelial to Mesenchymal Transition) using lung RNA from individual mouse cDNA template (*n* = 3). Details of the primer sequences used in this study are indicated in Supplementary Tables S2 to S4.

### Chromatin immunoprecipitation (ChIP) and OxiDIP assays

ChIP assays were performed using ChIP-IT Express kit with minor modifications. Briefly, cells grown in 15 cm plate were fixed with 1% formaldehyde for 10 min at room temperature and stopped by glycine buffer. Cells were collected and resuspended with hypotonic lysis buffer (10 mM HEPES, pH7.9, 1.5 mM MgCl_2_, 10 mM KCl) for 30 min on ice to remove cytoplasm protein. To the swollen cells in lysis buffer, 0.6% IGEPAL CA-630 was added, vortexed vigorously for 10 s and centrifuged (10 000 × *g*, 30 s). The nuclei pellet was resuspended with 50 mM Tris–HCl, 5 mM CaCl_2_ and digested with 1 μl Micrococcal Nuclease (2 × 10^3^ gel unites/μl) at 37°C for 15 min. Digestion was stopped by adding 10 μl of 0.5 M EDTA. Pellet nuclei by centrifugation at 16 000 × *g* for 1 min at 4°C and resuspend nuclear pellet in shearing buffer (50 mM Tris–HCl, pH 7.4, 150 mM NaCl, 5 mM CaCl_2_, 0.25% deoxycholic acid, 1% NP-40, 1mM EDTA, 0.1% SDS). Chromatin was sheared to yield 200–1000 bp of DNA. The sheared chromatin was centrifuged and carefully transfer supernatant for ChIP reaction with targeted antibody at 4°C overnight. 25 μl of protein A/G magnetic beads were added to pull down antibody-protein-DNA complex for 3 h at 4°C. This was spun briefly, washed and chromatin was eluted for qRT-PCR analysis. To test OGG1 enrichment, 4 μg of Flag-OGG1 was transfected into hSAECs per 10-cm petri dish, and anti-Flag (1:100) was used in the immunoprecipitation reaction.

OxiDIP assays to analyze 8-oxoG enriched DNA loci were performed as previously described (11,12) with modifications. After digestion with Micrococcal Nuclease, DNA was purified by phenol/chloroform. 4 μg of fragmented DNA was denatured and immuno-precipitated overnight at 4°C with 2 μl of 8-oxoG antibody in a final volume of 100 μl IP reaction (110 mM NaH_2_PO_4_, 110 Mm NaH_2_PO_4_ pH 7.4, 150 mM NaCl, 0.05% Triton X-100, 100 mM Tris–HCl pH 8.0, 0.5 M EDTA pH 8.0). Then, 25 μl of protein A/G magnetic beads were added for 3 h at 4°C, under constant rotation, and washed three times with 1 ml of washing buffer (110 mM NaH_2_PO_4_, 110 mM Na_2_HPO_4_ pH 7.4, 150 mM NaCl, 0.05% Triton X-100). All the steps of OxiDIP protocol, including the washes of the immunocomplexes, were carried out with deferoxamine mesylate (DFO, 100 μM) and phenyl-alpha-tert-butyl nitrone (PBN, 100 μM) to prevent oxidation of DNA during sample preparation. Experiments were performed in biological triplicates.

### Co-immunoprecipitation (co-IP) assay, his-tag pulldown and western blot (WB)

The association between OGG1 and phosphorylated SMAD3 (pSMAD3) in nuclear compartments was shown by Co-IP assays using modifications of a previously described method (35). Flag-OGG1 expressing hSAECs were immunoprecipitated with anti-Flag and immunoblotted with anti-pSMAD3. Furthermore, immunoprecipitation was also performed with anti-pSMAD3 and immunoblotted with anti-Flag. All steps were performed on ice or at 4°C and ice-cold buffers supplemented with cOmplete Protease Inhibitors, PhosSTOP Phosphatase Inhibitor Cocktail, 1 mM DTT and 1 mM PMSF. The cell pellet was incubated in five volumes of hypotonic lysis buffer (10 mM HEPES, pH 7.9, 10 mM KCl, 1.5 mM MgCl_2_) for 15 min to allow for cells to swell, then IGEPAL CA-630 was added to a final concentration of 0.6% and cells were incubated for six further minutes to lyse the plasma membrane. Nuclei were pelleted at 10 000 × *g* for 30 s, washed once with ten volumes of hypotonic lysis buffer, and finally re-suspended in two volumes of Nuclear Lysis Buffer (NLB, 50 mM Tris–HCl pH 7.5, 100 mM NaCl, 50 mM KCl, 3 mM MgCl_2_, 10% glycerol, 0.1% Tween-20). The nuclear suspension was transferred to a needle syringe (26G × 3/8) and homogenized by performing 70 strokes. The nuclear lysate was first mounted in vortex for 30 min, then 250 U/ml of Benzonase Nuclease was added to digest nucleic acids for 45 min at room temperature. Reactions were stopped by adding 5 mM EDTA and 5 mM EGTA. The lysate was clarified by ultracentrifugation at 20 000 × *g* for 15 min. The protein concentration was assessed, and 1 mg of protein was used for overnight IP with the primary antibody in rotation. IP were further incubated for 3 h with 20 μl of Protein A/G Mix Magnetic Beads, washed five times with NLB, and resuspended in 2 × Laemmli buffer.

His-Tag Pulldown assay was performed according to the instructions of His-tagged Dynabeads™. Briefly, pre-washed magnetic beads (20 μl) were incubated with His-tagged SMADs (20 nM) in binding buffer (50 mM Sodium phosphate, pH 8.0, 300 mM NaCl, 0.01% Tween-20) for 5 min at RT. Bead/protein complexes were resuspended in Pull-down buffer (3.25 mM Sodium phosphate, 70 mM NaCl, 0.01% Tween-20) and mixed with increasing concentrations of GST-OGG1 for 10 min at RT. After washing extensively, the elution was used in WB for detection of OGG1.

Proteins separated by electrophoresis were transferred to polyvinylidene difluoride (PVDF) membranes. Membranes were blocked for 3 h at room temperature in TBST (0.05% Tween-20) supplemented with 5% non-fat milk. Primary antibody was incubated overnight at 4°C. Images were developed by Amersham™ Image 680. Housekeeping proteins were used as loading controls to determine the normalization factors. Application of the normalization factor to the protein of interest generated relative expression levels.

### Electrophoretic mobility shift assay

Binding reactions were carried out in 10 μl reaction buffer containing 10 mM HEPES (pH 7.9), 10 mM KCl, 1.5 mM MgCl_2_, 1 mM DTT and 5% glycerol. All reactions were carried out on ice: proteins in reaction buffer were preincubated for 30 min, and 20 nM of DNA 5’-end labeled to Cy5 were added for another 30 min. To prepare nuclei extracts (NE), CelLytic™ NuCLEAR™ Extraction Kit was used. For super shift assays, 1 μg of antibodies were added to the NE (2 μg) for 1 h prior to the addition of probe. For competition experiments, 100-fold molar excess of poly d(I-C) was added to the binding reaction. DNA–protein complexes were resolved in 6% polyacrylamide gels (0.5 × TBE, 5% glycerol) in 1 × Tris glycine (TG) buffer and run for 120 min at 150 V at 4°C.

### Murine model of lung injury

C57BL/6J mice, free of specific pathogens were obtained from The Jackson Laboratories (Bar Harbor, ME USA). In the lung injury model, mice were challenged intranasally (i.n.) with saline as control or TGFβ1 (100 ng) three times weekly under mild anesthesia. TH5487 (30 mg/kg) was added intraperitoneally (i.p.) in a 100 μl volume as described previously (20). Control animals received an equivalent volume of vehicle (5% DMSO, 10% Tween 80 in saline). Animal experiments were performed according to the NIH Guide for Care and Use of Experimental Animals and approved by the University of Texas Medical Branch (UTMB) Animal Care and Use Committee (Protocol Number: 0807044D).

For 4 weeks after treatments, mice were euthanized, and bronchoalveolar lavage fluid (BALF) samples were collected as previously published (32). Briefly, tracheae were cannulated, and lung lavage was performed by two instillations of 0.7 ml of ice-cold PBS. BALF samples were centrifuged (200 × *g* for 5 min at 4°C), and the resulting supernatants were stored at −80°C for further analysis. Cell pellets were subjected to cytocentrifuge (Shandon Cytospin 4 Cytocentrifuge; Thermo Scientific) and stained with Wright-Giemsa. Differential cell counts were performed by two independent investigators blinded to the samples.

Tissue embedding, cutting, H&E, Masson's Trichrome staining (MTS) were done by Anatomic Pathology Laboratory of UTMB. IF staining of paraffin-embedded mouse lung was performed following published protocols (36). Briefly, lung sections (4 μm) were deparaffinized in xylene and hydrated in ethanol (100–70%). Sections were washed with deionized water. Antigen unmasking was done with citrate buffer. Sections were washed in deionized H_2_O and blocked with 10% goat serum for 3 h at RT, followed by primary antibody overnight at 4°C. Sections were washed with TBS-Tween 20 (0.1%) buffer and incubated with goat secondary Ab conjugated with Alexa Fluor 488 or 568 for 1 h at RT in the dark. Sections were washed in TBS-Tween 20 (0.1%), incubated with DAPI, mounted on coverslips, visualized by microscopy. Fluorescent areas were quantified using ImageJ software (37). Using ImageJ, MTS was quantified as percentage of positive pixels. Five fields of view were captured from each section, and the average value was calculated for each mouse (*n* = 7).

### Statistics

Comparisons of two groups were analyzed by Student's *t* test. Results are displayed throughout as mean ± S.D. Repeated measures analysis of variance was used to determine variables over time or between treatments. *P* < 0.05 was considered statistically significant.

## RESULTS

### Expression of OGG1 stabilizes TGFβ1 induced mesenchymal state

**Figure 1.**
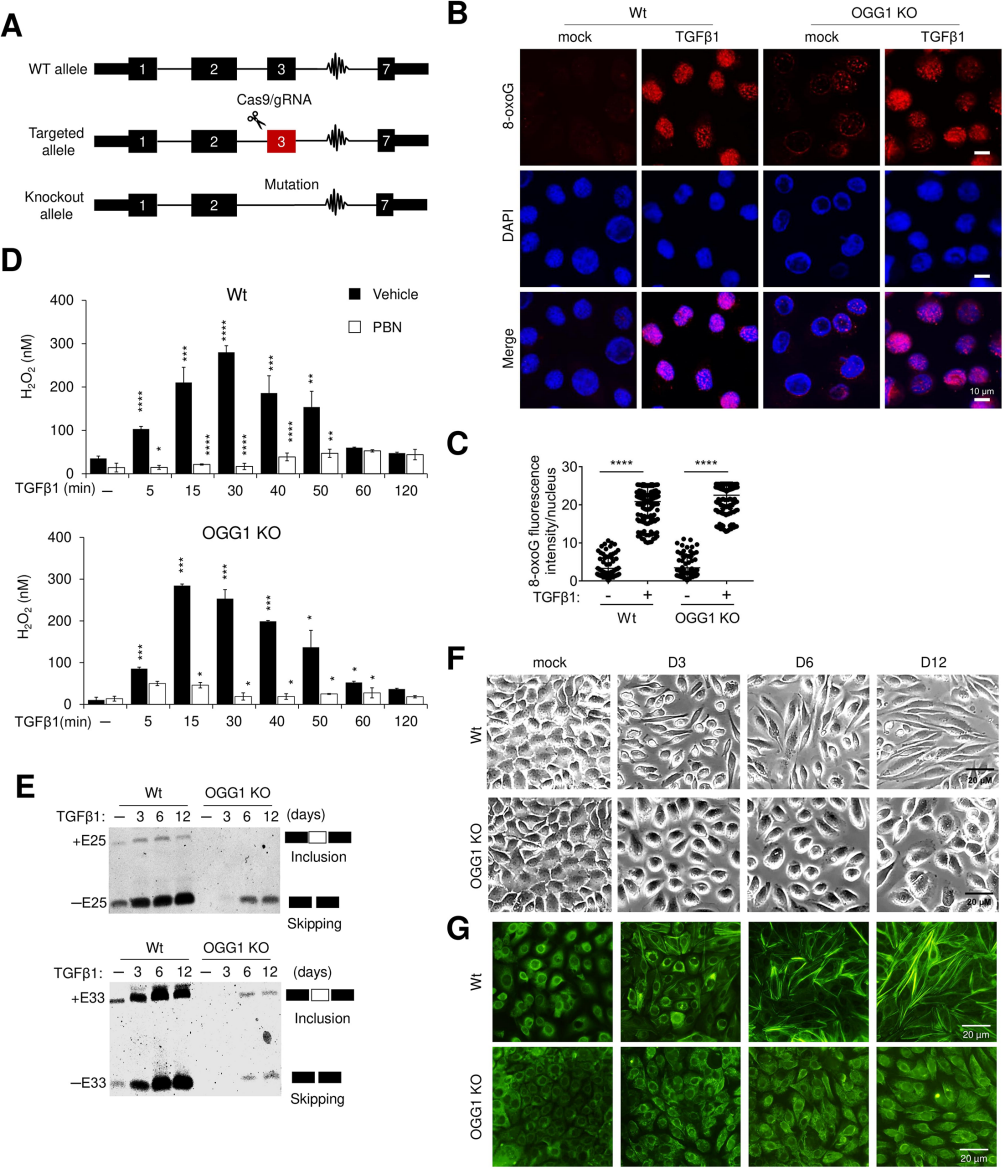
OGG1 is essential for EMT establishment in response to TGFβ1 exposure. (**A**) Diagram of CRISPR/Cas9 gene targeting used in OGG1 KO hSAECs. (**B**) Immunostaining of 8-oxoG in Wt and OGG1 KO hSAECs after TGFβ1 exposure for 1 h. Representative images are shown from three independent experiments in triplicate. Scale bars, 10 μm. (**C**) Quantification of 8-oxoG fluorescence intensity per nucleus from a representative experiment in (B) (≥300 nuclei were analyzed per condition). (**D**) Wt and OGG1 KO hSAECs were treated with TGFβ1 ± PBN for indicated times, and intracellular H_2_O_2_ levels were assessed using Amplex Red. *P < 0.05, **P < 0.01 and ***P < 0.005, by a two-tailed unpaired *t*-test. (**E**) Transcript analysis showing inclusion levels of *FN1* Exon 33 and exclusion of Exon 25 in Wt and OGG1 KO hSAECs. (**F**) Phase contrast microscopy presents morphological evidence of EMT in Wt, but not OGG1 KO hSAECs with TGFβ1 treatment for indicated days. (**G**) Wt and OGG1 KO hSAECs were challenged with TGFβ1 for indicated days. Actin filaments (F-actin) were stained with FITC-Phalloidin.

Since oxidative stress is generated by TGFβ1 signaling (38), we determined the magnitude and kinetics of oxidative DNA damage upon TGFβ1 exposure by measuring of 8-oxoG levels. We found that 8-oxoG levels are rapidly and significantly increased in response to TGFβ1, and this increase was prevented by pretreatment with the free radical scavenger N-tert-Butyl-α-phenylnitrone (PBN) (Supplementary Figure S1A, B). This result suggests that 8-oxoG damage-specific binding by OGG1 may be involved in TGFβ1 signaling. We utilized CRISPR/Cas9 technology to generate OGG1 specific knockout (KO) hSAECs (Figure 1A and Supplementary Figure. S1C, D). In both wild-type (Wt) and OGG1 KO cells, 8-oxoG levels (Figure 1B, C), H_2_O_2_ production (Figure 1D, Supplementary Figure S1E), oxidized proteins with carbonyl modifications (Supplementary Figure S1F) and nitrosative protein levels (Supplementary Figure S1G) were similarly increased in response to TGFβ1 exposure, indicating a functional ROS machinery in the absence of OGG1.

TGFβ1 is a potent inducer of developmental and fibrogenic EMT. To explore the function of OGG1 in type II EMT, we measured epithelial and mesenchymal markers in both Wt and OGG1 KO cells. In Wt cells, prolonged TGFβ1 treatment produced a loss of expression of the epithelial adhesion protein E-cadherin, and an increase in mesenchymal Vimentin (VIM), Fibronectin (FN1) and Collagen (COL1A1) levels, associated with redistribution of the mesenchymal regulator, Snail Family Transcriptional Repressor 1 (SNAI1), into nuclei. Importantly, these hallmarks of EMT-associated events are absent in OGG1 KO hSAECs (Supplementary Figure S2A, B). We also observed less incorporation of α-smooth muscle actin (αSMA) into stress fibers in *Ogg1*^−/−^ compared to *Ogg1*^+/+^ MEF cells (Supplementary Figure S2C). Attainment of a stable EMT results in secretion of alternative splice variants of extracellular matrix (ECM) proteins, including FN (39). Extra type Ⅲ domain A (EⅢA)-containing FN is necessary for pulmonary fibrosis to develop (39). Thus, we further examined the effect of OGG1 on FN alternative transcript expression using primers that span the splice sites of exon 25 (EⅢB) and exon 33 (EⅢA) (40). Transcripts for exon 33 included FN and exon 25 excluded FN variants are induced in a time-dependent manner in Wt cells, but both are very low in OGG1 KO cells (Figure 1E). Apart from using protein markers to define the EMT program, cell phenotypic plasticity is key to characterize this process (41). We observe that TGFβ1 treated Wt cells progressively lose their cobblestone epithelial appearance in monolayer cultures to adopt a spindle-shaped, fibroblastic morphology (Figure 1F) and assemble bundles of actin filaments throughout the cytoplasm (Figure 1G). The elongated fibroblastic appearance of these features is prevented in OGG1 KO cells (Figure 1F, G). Altogether, these data suggest that OGG1 is functionally required for the development of EMT phenotypic changes.

### 8-oxoG functions as an epigenetic mark bound by OGG1 in active promoters of fibrotic genes

**Figure 2.**
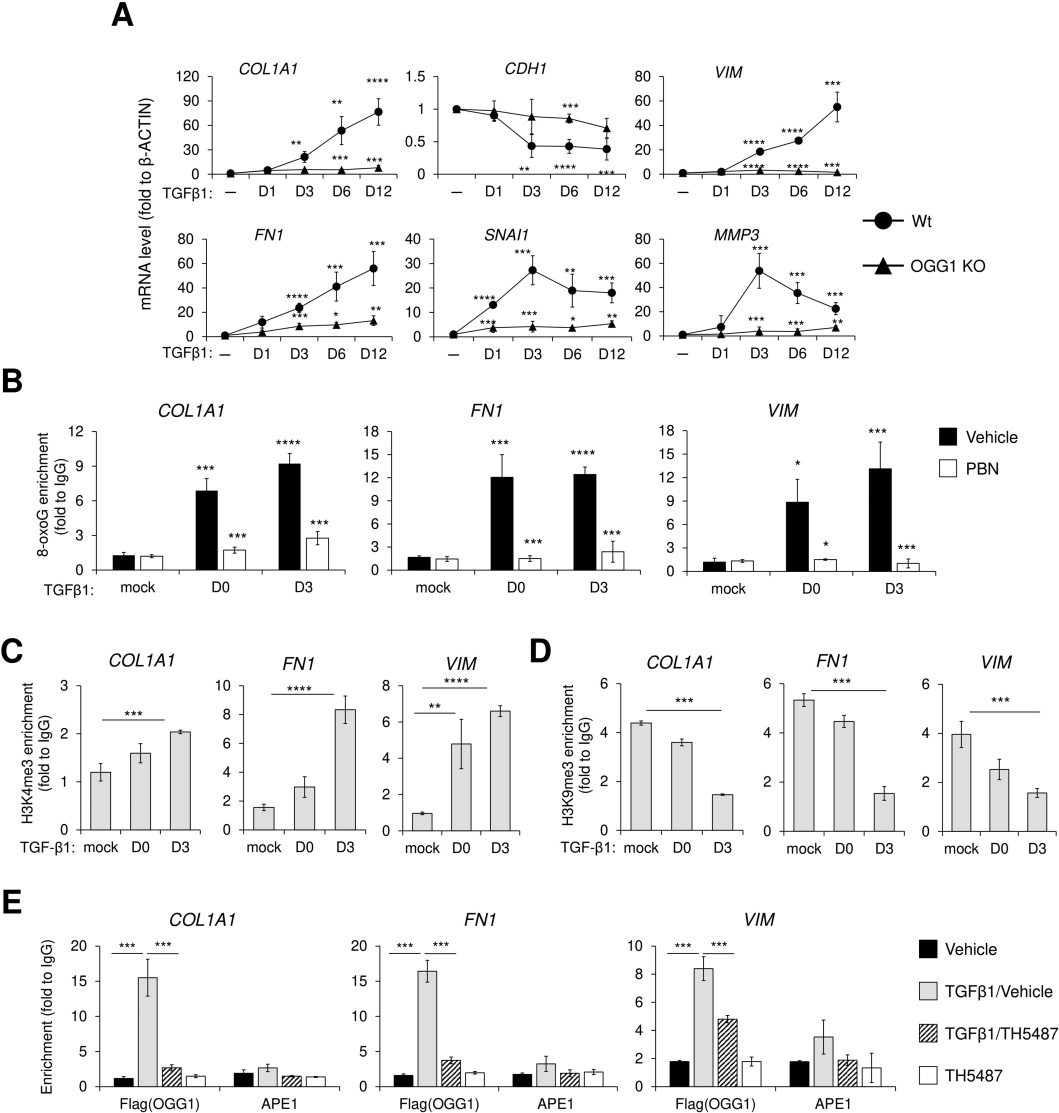
8-oxoG load correlates with epigenetic marks for open chromatin in active transcription. (**A**) qRT-PCR analyses of EMT genes in Wt and OGG1 KO hSAECs with TGFβ1 treatment for indicated days. (B–D) hSAECs were mock or TGFβ1 treated, DNA was extracted and OxiDIP assays were performed using antibody against 8-oxoG (**B**), and chromatin was immunoprecipitated with antibodies to H3K4me3 (**C**) and H3K9me3 (**D**). (**E**) TH5487 inhibits OGG1 enrichment on chromatin. Flag-OGG1 expressing hSAECs were pretreated with vehicle or TH5487 for 1 h, followed by TGFβ1 addition for 1 h. ChIP experiments were performed using antibodies against Flag and APE1. In B–E, fold change is normalized to IgG by qRT-PCR. Data are presented from three independent experiments in triplicate. *P < 0.05, **P < 0.01 and ***P < 0.005, by a two-tailed unpaired *t*-test.

Cell plasticity in type II EMT is the result of temporally coordinated patterns in gene expression (42). To document the link between OGG1 and transcriptional regulation in type II EMT, we conducted OGG1 loss-of-function experiments with CRISPR/Cas9, non-toxic pharmacologic inhibitors (Supplementary Figure S3A, B) and small interfering RNA (siRNA) technologies. In OGG1 proficient cells, TGFβ1-induced mesenchymal gene expression in a pattern that increased over a period of 12 days, as shown by *Collagen1A1* (*COL1A1*), *FN1*, *VIM, Matrix Metallopeptidase 3* (*MMP3*) and *SNAI1* mRNA level (Figure 2A). Similarly, in Wt cells, TGFβ1-induced a loss of *E-cadherin* (*CDH1*). By contrast, cells lacking functional OGG1 had markedly blunted mesenchymal gene induction and relative preservation of *CDH1* expression (Figure 2A, Supplementary Figure S3C–F). We also tested OGG1 expression over this period, showing accumulated OGG1 mRNA level after chronic exposure (12 days) (Supplementary Figure S4A). This result indicates that the regulatory function of OGG1 may act through increased expression levels. Because EMT is a dynamic process, we used terminally differentiated fibroblast cells (MRC5), which constitutively express *COL1A1*, *FN1* and *VIM*, to further define OGG1 function in mesenchymal reprogramming. Two different inhibitors that disrupt 8-oxoG-OGG1 interaction have been shown to decrease inflammation-induced cellular effects, TH5487 (20) and SU0268 (31,43). TH5487 and SU0268 treatment of MRC5 over a period of 3 days resulted in decreased *COL1A1*, *FN1* and *VIM* mRNA levels (Supplementary Figure S4B), revealing that OGG1 also regulates fibrotic gene expression at steady state. TH5487 also inhibited TGFβ1-induced fibroblast-like appearance in hSAECs (Supplementary Figure S5A), whereas administration of inactive analog TH2840 or inhibitor O8, that do not interfere with 8-oxoG-specific binding, had no effect (Supplementary Figure S5B). Altogether, these data suggest that OGG1 is critical for both inducible and stable fibrotic gene transcription.

We next assessed how 8-oxoG orchestrates promoter function following its recognition by OGG1. Previous studies have shown that ROS-mediated oxidative base modifications occur in the context of transcriptionally active nucleosomes sensitive to nuclease digestion (44). Therefore, we examined micrococcal nuclease-derived nucleosome fractions and the distribution of 8-oxoG. We found 8-oxoG enrichment on *COL1A1* (–350/–176), *FN1* (–587/–419) and *VIM* (–324/–142) to be significantly increased after repeated TGFβ1 exposure, with this process being ROS-dependent (Figure 2B). Micrococcal nuclease-sensitive nucleosomes bear some relation to the state of open chromatin organization (44). We next investigated whether 8-oxoG generation is related to chromatin state. We found active promoter marker, H3K4me3, to be significantly increased (Figure 2C), while heterochromatin marked by H3K9me3 was significantly decreased after TGFβ1 exposure (Figure 2D), which indicates that higher order chromatin compaction protects DNA integrity from oxidative damage. Examination of OGG1 binding at individual gene promoters revealed that OGG1 occupies sites with 8-oxoG, with this binding pattern abrogated by TH5487 (Figure 2E). The enrichment of apurinic/apyrimidinic endoDNase 1/redox factor1 (APE1/Ref1) was under detectable levels by ChIP assays (Figure 2E), suggesting its dispensable role in initiation of gene expression. This has been further tested with APE Inhibitor Ⅲ, which does not interfere with its binding to the AP site but inhibits endonuclease activity (24). The mRNA levels of selected EMT genes are not significantly changed with APE Inhibitor Ⅲ, while blocking the redox factor activity of APE1/Ref1 with E3330 (45) significantly decreases mRNA levels (Supplementary Figure S6). This suggests a role of APE1/Ref1 as a redox factor needed for EMT gene expression.

### OGG1 dependent recruitment of SMAD complexes to fibrotic gene promoters in chromatin

**Figure 3.**
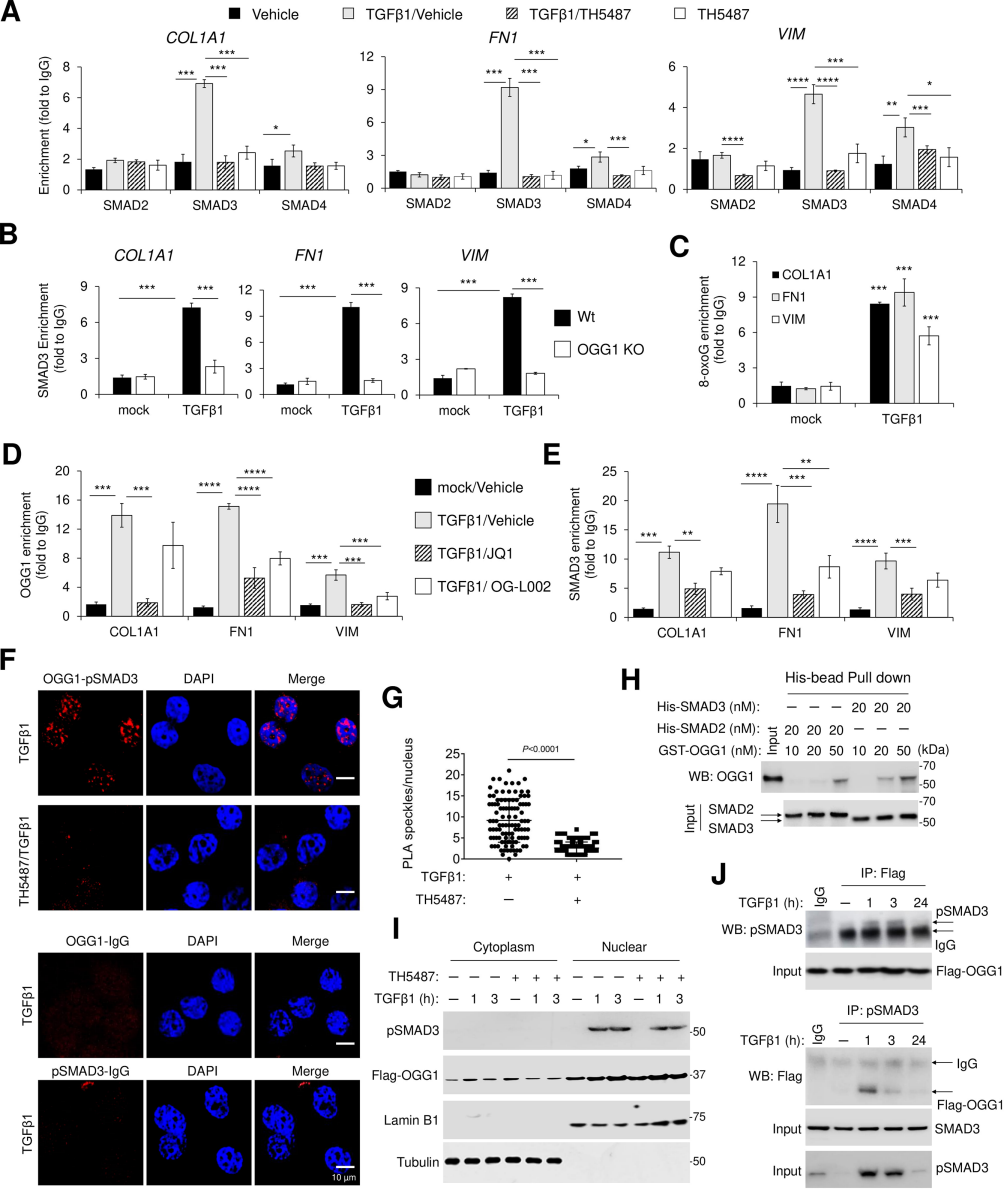
TGFβ1 induced OGG1 co-occupancy with SMAD3 on fibrotic gene promoters. (**A**) Enrichment of SMAD2, SMAD3 and SMAD4 in promoter region of *COL1A1*, *FN1* and *VIM*. hSAECs were pretreated with vehicle or TH5487 for 1 h, followed by TGFβ1 addition for 1 h. ChIP experiments were performed using antibodies against SMAD2, SMAD3 and SMAD4. (**B**) Wt and OGG1 KO cells were incubated with TGFβ1, and ChIP assays were performed using antibody to SMAD3. (**C**) SMAD3 ChIP-ed DNA contains 8-oxoG. ChIP assays were performed using SMAD3 ab from the TGFβ1 exposed hSAECs, DNA is eluted and immunoprecipitated with anti-8-oxoG Ab. hSAECs were pretreated with JQ1 or OG-L002 for 1 h following by adding TGFβ1 for 1 h, chromatin was immunoprecipitated with antibodies to Flag (OGG1) (**D**) and SMAD3 (**E**). In A–E, fold enrichment is normalized to IgG by qRT-PCR. Data are presented from three independent experiments in triplicate. *P < 0.05, **P < 0.01 and ***P < 0.005, by a two-tailed unpaired *t*-test. (**F**) Proximity ligation assays (PLA) for OGG1 and phosphorylated SMAD3 (pSMAD3) in hSAECs maintained in the presence of TGFβ1. Representative images are shown from three independent experiments in triplicate. Flag or pSMAD3 antibodies paired with IgG were used as negative control. Scale bars, 10 μm. (**G**) Quantification of PLA speckles in nucleus compartment. For each experimental condition, a minimum of 100 cells from a total of 3 independent experiments were analyzed. *P* < 0.0001, by a two-tailed unpaired *t*-test. (**H**) *In vitro* His-Tag pulldown of His-SMAD2 and His-SMAD3 with GST-OGG1. (**I**) Cytoplasmic and Nuclear fractions were analyzed by WB showing pSMAD3 and Flag-OGG1 levels. Lamin B1 indicates nuclear compartment, and αTubulin indicates cytoplasm compartment. (**J**) Co-IP assays showing the interaction between OGG1 and pSMAD3 in nuclear compartment. Isotype IgG serves as negative control, which incubated with mixed lysates from mock and TGFβ1 exposed ones. Representative images are shown from three independent experiments. For figure (I) and (J), full membranes are shown in Supplementary Figure S7.

TGFβ1 signaling activates SMAD transcription factors, where they bind to regulatory regions of target genes to elicit new programs of gene expression (46). ChIP analysis shows the enrichment of SMAD2, SMAD3 and SMAD4 in *COL1A1*, *FN1* and *VIM* promoters upon TGFβ1 exposure. We observe that OGG1 functional absence results in significantly lower levels of SMAD3 and partial SMAD4 occupancy, as shown by TH5487 addition and by using OGG1 KO cells (Figure 3A, B). If 8-oxoG serves as an epigenetic-like mark in TGFβ1-induced gene expression, then SMAD3 should co-occupy DNA sites with 8-oxoG. Using a two-step affinity enrichment in ChIP, first using SMAD3 Ab to capture chromatin, followed by elution and pulldown with an 8-oxoG Ab, we show that SMAD3 and 8-oxoG are both enriched at the *COL1A1*, *FN1* and *VIM* promoters after TGFβ1 treatment (Figure 3C). The link between the degree of chromatin compaction and gene transcription is well established (47,48). Bromodomain containing protein 4 (BRD4) is a histone acetyltransferase (HAT) that actively mediates chromatin decompaction by evicting nucleosomes from chromatin (49). Lysine-specific histone demethylase (LSD1) is a putative generator of ROS through demethylation of histone H3 (50). Nuclear factor kappa B (NF-κB) directly binds to key EMT gene promotors and activates their expression by forming elongation complexes with BRD4 (51). Inhibition of BRD4, LSD1 and NF-κB function using JQ1, OG-L002 and BMS345541, respectively, showed decreased mRNA levels of EMT genes (Supplementary Figure S6). Since oxidative stress redistributes OGG1 to euchromatin regions (33,52), it is tempting to speculate that the enrichment of OGG1 and transcription factors in chromatin is affected by chromatin remodeler. Treatment with JQ1 significantly blocks OGG1 and SMAD3 enrichment to *COL1A1*, *FN1* and *VIM* gene promoter, with partial effects seen with OG-L002 (Figure 3D, E). These results highlight that TGFβ1-driven chromatin reconfiguration and promoter accessibility are necessary for OGG1 function via 8-oxoG.

TGFβ1 signaling activates nucleocytoplasmic shuttling of the SMADs and SMAD3 phosphorylation (53). To determine whether OGG1 interacts with phosphorylated (p) SMAD3, we examined the association by proximity ligation assay (PLA) *in cellulo*. TGFβ1 exposure induces a significant number of PLA foci in the nucleus, while TH5487 pretreatment significantly decreased it (Figure 3F, G), suggesting OGG1 interacts with pSMAD3 while recognizing genomic substrates. *In vitro* pull-down assay shows that OGG1 is in complex with SMAD2 and SMAD3 (Figure 3H and Supplementary Figure S7C). To confirm this interaction *in cellulo*, we first isolated nuclear extracts (NE) and showed that the OGG1 inhibitor TH5487 has no effect on SMAD3 phosphorylation and its nuclear translocation in response to TGFβ1 treatment (Figure 3I). NE co-immunoprecipitation (Co-IP) assays were performed showing pSMAD3 interaction with ectopically expressed OGG1 (Flag-OGG1) after TGFβ1 treatment. (Figure 3J). Taken together, these data indicate that OGG1 interacts with activated SMAD3 during TGFβ1 signaling.

### OGG1 directly recruits SMAD3 to adjacent SMAD binding elements (SBEs)

**Figure 4.**
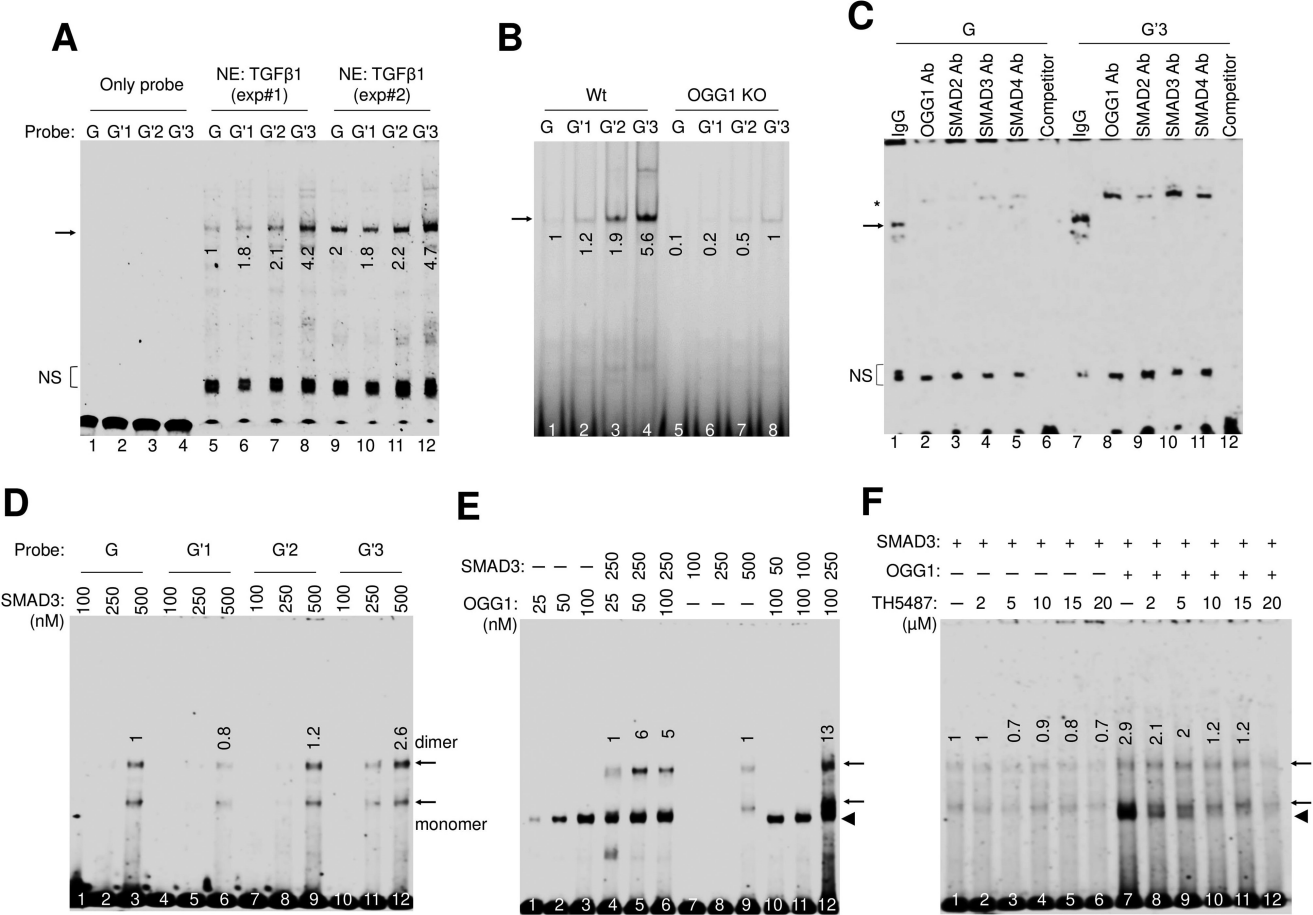
OGG1 is essential for the formation of SMAD3-SBE complex under oxidative stress. (**A**) SMAD–DNA complex formation modulated by 8-oxoG position in OGG1 proficient NE. NE from two independent experiments were incubated with control (G) and 8-oxoG modified oligo (G'1, G'2, G'3), and EMSA was performed. (**B**) OGG1 deficiency abrogates SMAD binding to SBE containing oligo. Wt and OGG1 KO cells were pre-exposed with TGFβ1 for 1 h, and NE were prepared to perform EMSA as in (A). (**C**) SMAD–DNA complex contains OGG1. hSAECs treated with TGFβ1 for 1 h and extract nuclear proteins. NE was incubated with antibody against OGG1, SMAD2, SMAD3 or SMAD4, and performed EMSA using G and G’3 probe. IgG and cold competitor served as negative control. Star indicates super-shifted complexes. In A–C, arrows show SMAD-DNA complex. NS indicates non-specific. (**D**) EMSA shows recombinant SMAD3 binds to control (G) and 8-oxoG-modified (G'1, G'2, G'3) oligo. (**E**) OGG1 facilitates SMAD3 binding to G'3 probe and analyzed by EMSA. (**F**) Recombinant OGG1 and SMAD3 as indicated were incubated in the presence of increasing concentration of TH5487, and G'3 probe was used to test DNA–protein complex formation with EMSA. In D–F, arrow indicates migrating complexes containing SMAD proteins. Triangle indicates OGG1–DNA complex. Fold change in intensity is labeled with numbers. Representative image shows gel shift from at least three independent experiments.

If OGG1 and pSMAD3 were to form a physical complex, we would expect OGG1 and pSMAD3 to co-occupy the same sites under oxidative stress. It was identified that CAGAC motifs are predominantly used for SMAD3/4-dependent transcriptional activation (54). To evaluate the effect of OGG1 bound to 8-oxoG on SMAD DNA occupancy on SBEs, we synthesized a series of oligonucleotides that correspond to the sequence from *VIM* (–257/–200) containing CAGAC motif, including the 3 different probes (G'1–G'3) that containing single 8-oxoG at 5’ of GGG (one in the sense strand, and two in the anti-sense strand), and the normal non-substituted probe (G) (Table 1). We carried out gel shift assays and show extensive G probe shifts in TGFβ1 treated nuclear extracts (NE) to mock (Supplementary Figure S8A), suggesting TGFβ1 signaling activates SMAD translocation to the nucleus. Compared with G probe, with an 8-oxoG base placed at 5 bp upstream SBE in the antisense strand (G’3), the protein-DNA shift becomes significantly increased (Figure 4A). The SMAD–DNA complex was significantly lower in NE from OGG1 KO cells (Figure 4B, Supplementary Figure S8B), and TH5487 interferes with TGFβ1-inducible DNA–protein complex in a concentration dependent manner (Supplementary Figure S8C), indicating that OGG1 is required for the formation of inducible DNA–protein complexes. Antibodies against OGG1, SMAD2, SMAD3 and SMAD4 yield a super shift of the complex, not seen with IgG (Figure 4C), indicating that TGFβ1-inducible DNA-protein complex is composed of OGG1, SMAD2, SMAD3 and SMAD4. OGG1 antibody shifted G probe to a level through protein-protein interaction. Conversely, competitors in excess abolish the shifts in G and G'3 probes (Figure 4C), suggesting SMAD binding to these motifs was specific. These results are consistent with ChIP assays and again suggest that 8-oxoG recognition by OGG1 in close proximity to SBE would facilitate SMADs-DNA complex formation.

**Table 1.** Sequences of oligonucleotides used for electromobility shift assay. X indicates the substitution of 2′-deoxyguanine by 8-oxoG

Oligomer duplex	Sequence of *VIM (–257/–200)*
G	3′-GGGGTCCCACTCGGGTCGAGTCTGATAGTAGGCCTTTCGGGGGTTTTCAGGGTCGGG-5′
	/CY5/5′-CCCCAGGGTGAGCCCAGCTCAGACTATCATCCGGAAAGCCCCCAAAAGTCCCAGCCC-3′
G'1	3′-GGGGTCCCACTCGGGTCGAGTCTGATAGTAGGCCTTTCGGGGGTTTTCAGGGTCGGG-5′
	/CY5/5′-CCCCAXGGTGAGCCCAGCTCAGACTATCATCCGGAAAGCCCCCAAAAGTCCCAGCCC-3′
G'2	3′-GGGGTCCCACTCGGGTCGAGTCTGATAGTAGGCCTTTCGGGGGTTTTCAGGXTCGGG-5′
	/CY5/5′-CCCCAGGGTGAGCCCAGCTCAGACTATCATCCGGAAAGCCCCCAAAAGTCCCAGCCC-3′
G'3	3′-GGGGTCCCACTCGGXTCGAGTCTGATAGTAGGCCTTTCGGGGGTTTTCAGGGTCGGG-5′
	/CY5/5′-CCCCAGGGTGAGCCCAGCTCAGACTATCATCCGGAAAGCCCCCAAAAGTCCCAGCCC-3′

To further explore the effects of OGG1 on SMADs binding, we used recombinant, full-length SMAD2 and SMAD3 proteins in gel shift experiments. Only 8-oxoG containing probes (G'1 to G'3) are recognized by OGG1 (Supplementary Figure S8D). SMAD3 binding capacity to the four probes does not show significant differences between non-substitute and 8-oxoG containing probes at the highest concentration used in these studies (Figure 4D), which indicates that 8-oxoG alone in the DNA duplex does not lead to SMAD3-recognizable conformational changes. Preincubation of OGG1 with SMAD3 increased SMAD3-DNA complex formation in a concentration dependent manner (Figure 4E). Since TH5487 is a selective molecule that inhibits OGG1 binding to 8-oxoG, SMAD3 binding should not be affected by TH5487, with decreased SMAD3-G'3 shift detected in the presence of OGG1 (Figure 4F). SU0268 also decreased SMAD3 binding to the G'3 probe in the presence of OGG1, but not TH2840 or O8 (Supplementary Figure S8E). Thus, OGG1 is necessary for SMAD3 binding to DNA containing SBEs, which are dependent on the 8-oxoG position. Only OGG1, but not double-strand uracil-DNA glycosylase (UDG), 8-oxo-7,8-dihydrodeoxyguanosine triphosphatase (MTH1) or NEIL2 bound G’3 probe and increased SMAD2 and SMAD3 binding to SBE (Supplementary Figure S8F, G). Thus, OGG1 formed a selective complex with 8-oxoG that significantly increases SMADs binding to SBEs containing DNA. SMAD3 bound to SBE had no effect on OGG1 mediated base removal (Supplementary Figure S8H). OGG1 loss-of-function does not affect TFs expression and nuclear translocation, as shown with SMAD3 and NF-κB (Supplementary Figures S9 and S10). The mechanism highlighted herein may be explained by some of our previous work demonstrating that OGG1 promotes NF-κB activity by increasing DNA occupancy on the κB site (21). Together, these studies suggest a role of OGG1 in facilitation of SMAD binding to targeted gene loci without affecting SMADs expression, phosphorylation, or translocation.

### Inhibition of OGG1 initiated BER reduces the fibrotic response in murine lung

**Figure 5.**
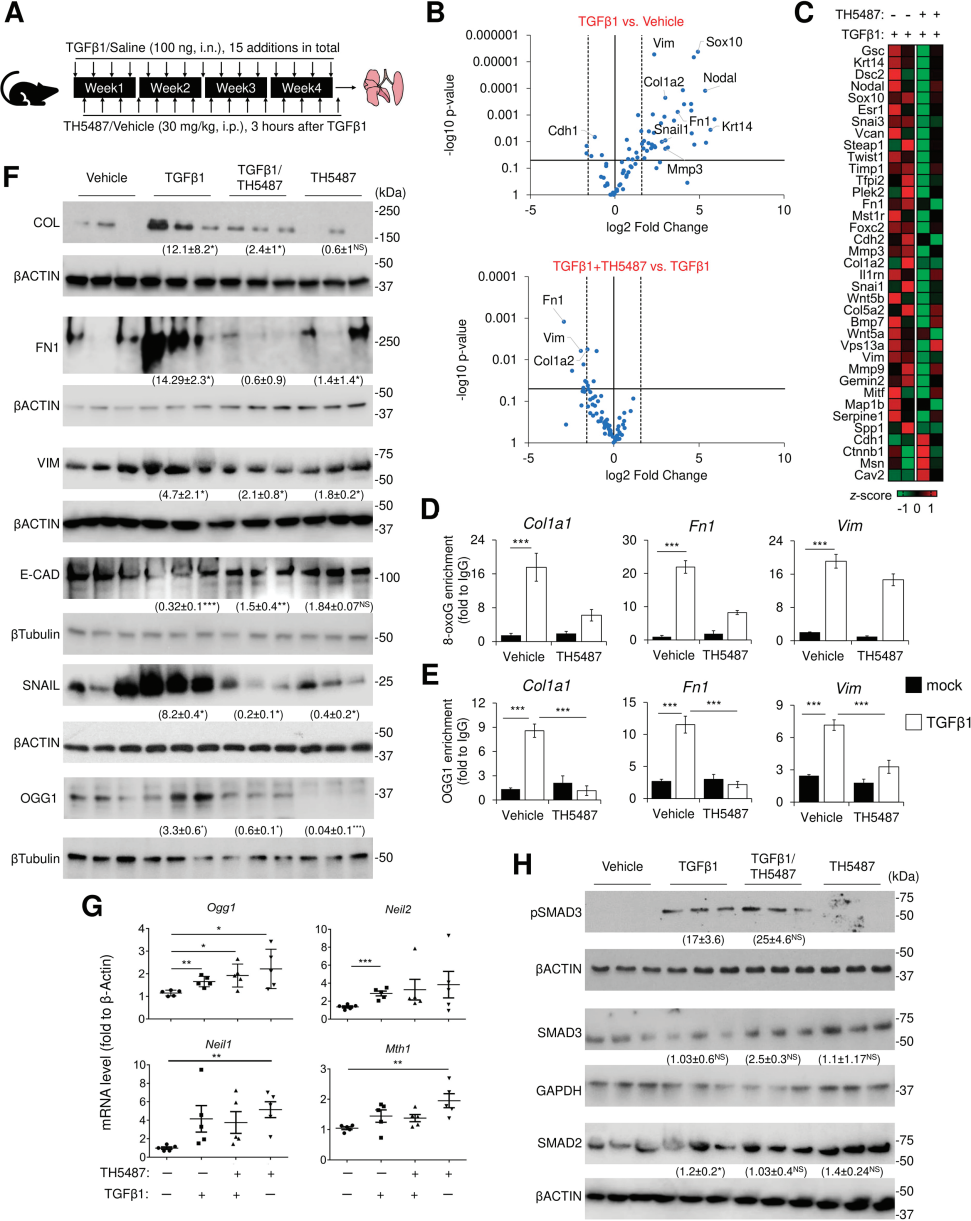
EMT pathway targeted PCR array reveals gene expression regulated by OGG1. (**A**) Experimental outline for murine model subjected to TGFβ1 and treated with vehicle or TH5487 (*n* = 7 in each group). (**B**) Volcano plots show mRNA level changes from murine lung (three individual mice). (**C**) Heat map of regulated transcripts from murine lung (two individual mice). Scale indicates *z*-score where red is high, and green is low. (**D**) Analysis of 8-oxoG enrichment on *Col1a1*, *Fn1* and *Vim* promoter from murine lung. (**E**) ChIP analyses of OGG1 enrichment on *Col1a1*, *Fn1* and *Vim* promoter from murine lung. (**F**) Expression of epithelial and mesenchymal markers in murine lung were analyzed by immunoblotting and quantified by normalization to housekeeping proteins. Full membrane blots are shown in Supplementary Figure S12. Data are mean ± SD from three individual mice. (**G**) mRNA level of DNA repair protein from murine lung was quantified by qRT-PCR and normalized to βActin. Data are presented as mean ± SD from five individual mice. (**H**) Levels of SMAD3 phosphorylation, total SMAD3 and SMAD2 from murine lung were analyzed by immunoblotting. Data are presented mean ± SD from three individual mice. In (D–H), *P < 0.05, **P < 0.01 and ***P < 0.005, by a two-tailed unpaired *t*-test.

Our results obtained from cultured cells highlight 8-oxoG-specific binding by OGG1 as a signaling node during transcription. We next assessed whether OGG1 can regulate fibrotic pathogenesis using an *in vivo* murine model. Mice received TH5487 or vehicle through i.p. and were administered TGFβ1 i.n. (Figure 5A). We performed 96 PCR arrays specifically targeting the EMT pathway using lung cDNA from 3 individual mice. We identified a population of EMT genes that are changed by TH5487, including *Col1a2*, *Fn1* and *Vim*, as well as direct TGFβ1/Smad3-targeted genes encoding Serine (or cysteine) peptidase inhibitor, clade E, member 1 (Serpine1), Keratin 14 (Krt14, which is absent in distal airways of healthy lungs, but increased specifically in distal airways and alveolar regions of Idiopathic pulmonary fibrosis lungs) (55), Nodal (ligand of various TGFβ1 receptors), SRY-box containing gene 10 (Sox10, transcription factors involved in the regulation of embryonic development and in the determination of the cell fate), with no up-regulation of these genes observed in TH5487-treated murine lungs (Figure 5B, C, Supplementary Figure S11A, B, Supplementary Table 5). Enrichment analysis in transcriptional regulatory relationships showed that TH5487 regulated genes are substantially regulated by Smad3, Etv4, NF-κB and other TFs (Supplementary Figure S11C). Gene ontology clusters indicated that the regulated pathways are significantly involved in EMT and ECM organization networks (Supplementary Figure S11D, E). Indeed, we observed that collagen deposition is reduced by TH5487 in lung tissue (Supplementary Figure S11F). In murine lung, high levels of 8-oxoG correlate with OGG1 enrichment to fibrotic gene promoters (Figure 5D-E). Consistent with array and individual qRT-PCR data, WB using lung lysates showed that TH5487 treatment decreased protein levels with mesenchymal characteristics and increased those with epithelial characteristics like E-CAD (Figure 5F). Moreover, TH5487 decreases OGG1 level via NEDD4-like E3 ubiquitin protein ligase-mediated degradation (56). In addition, the mRNA expression of *Ogg1* increased, while *Neil1* and *Mth1* are not significantly changed in response to TGFβ1 (Figure 5G). Note, there was an increase in *Neil2* mRNA levels, which is explained by its promoter containing *cis* elements for stress responsive transcription factor (57). SMAD3 phosphorylation was enhanced in lung treated with TGFβ1, but not significantly altered by TH5487 treatment (17 ± 3.6 compared with 25 ± 4.6 in TGFβ1 treated mice), suggesting inhibition of OGG1 binding to genomic substrates has no effect on SMAD3 phosphorylation (Figure 5H). Together, TH5487 administration *in vivo* manifests as lower levels of transcripts associated with epithelial-mesenchymal interactions, suggesting that OGG1 triggers distinct cell-behaviors in the lung.

**Figure 6.**
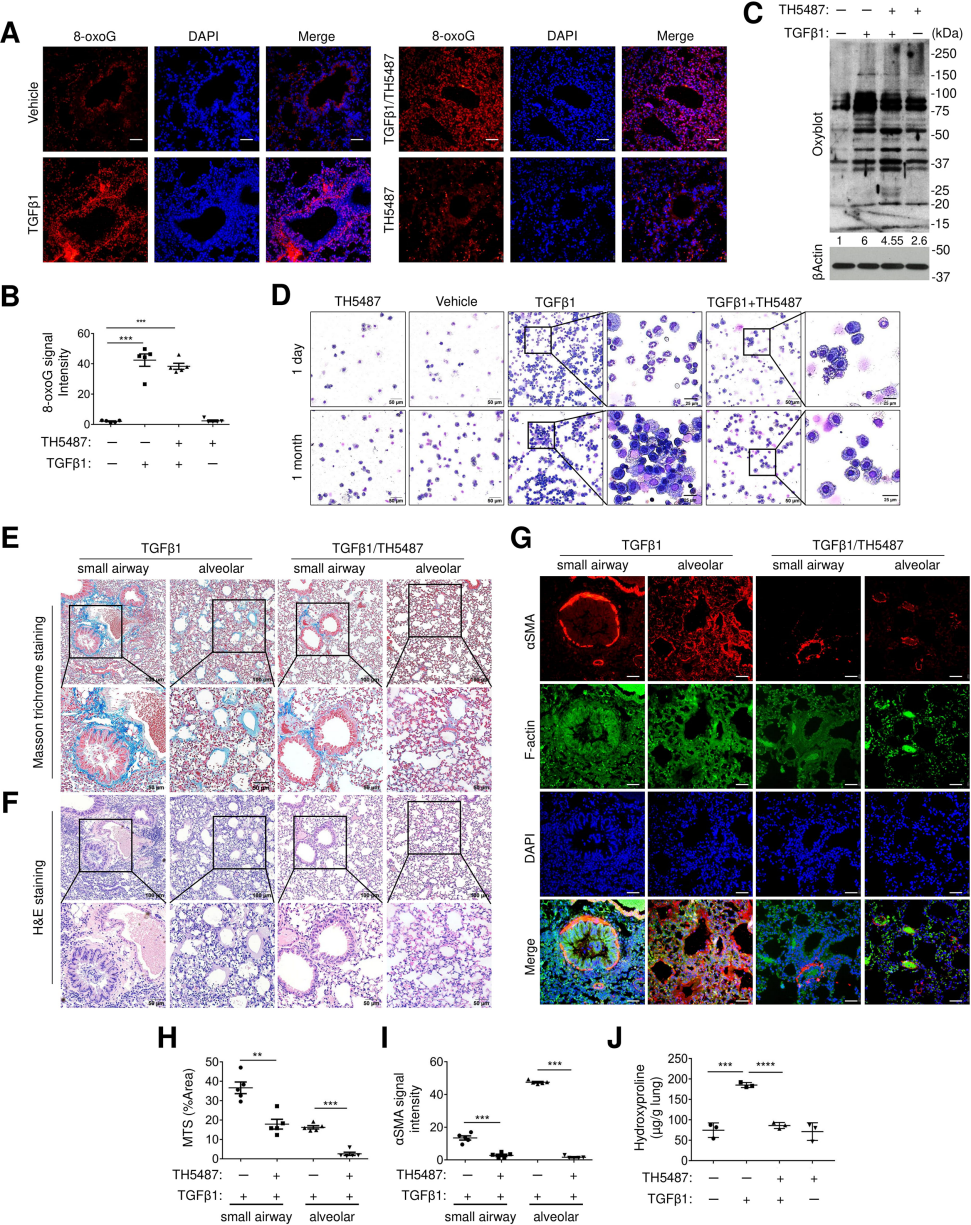
Inhibition of OGG1-8-oxoG interaction suppresses tissue remodeling. (**A**) Representative images of immunohistochemistry of 8-oxoG in murine lung. Sections were counterstained with DAPI to detect nuclei. Scale bars, 50 μm. (**B**) Proportion of nucleus 8-oxoG-positive cells in airway and alveoli were quantified by image analysis (*n* = 5). (**C**) Protein carbonyl levels in murine lung (*n* = 7) were analyzed by western blotting (Oxyblot). (**D**) Giemsa-Wright stained cytospin from BALF. Scale bars, 50 μm (low magnification) and 20 μm (high magnification). (**E**) Masson's trichrome stain (MTS) exhibits collagen deposition around small airway and alveolar wall. (**F**) H&E staining shows inflammatory cell infiltration around airways. (**G**) *In situ* IF staining showed αSMA expression was localized around airways and blood vessels in lung tissue. Scale bars, 50 μm. (**H**) MTS and (I) αSMA immunostaining staining with the quantifications. Data are presented as mean ± SD from five individual mice. Representative images are shown from seven individual mice. (**J**) Measurement of hydroxyproline level in murine lung. Data are presented as mean ± SD from three individual mice. **P < 0.01 and ***P < 0.005, by a two-tailed unpaired *t*-test.

Given that oxidative stress in response to TGFβ1 exposure could induce signaling in lung pathology, lung sections prepared from mice exposed to TGFβ1 ± TH5487 were stained for the oxidative DNA marker, 8-oxoG. Exposure to TGFβ1 markedly increased nuclear 8-oxoG staining intensity and the number of positively stained cells in the small airway and alveoli (Figure 6A, B). While in vehicle and TH5487-treated mice, diffuse 8-oxoG staining was seen in perinuclear regions (Figure. 6A, B). TGFβ1 increased oxidatively modified protein levels in the lung tissue as determined by Oxyblot analysis but were not significantly changed in the presence of TH5487 (Figure. 6C). BALF cyto-spins from TGFβ1-exposed mice (1 day) showed increases in neutrophil influx into the airways, while in TGFβ1 exposure for 1 month shows accumulation of macrophages (Figure. 6D). These proportions are decreased in the presence of TH5487, suggesting TH5487 can be used in treating both acute (20) and chronic inflammation (58). Consistent with these observations in lung tissue, cellular 8-oxoG staining from BALF showed no difference in nuclear intensity but did display a decreased cell number with TH5487 administration (Supplementary Figure S13A). Histological examination of lung tissues using Masson's Trichrome (Figure 6E and H, Supplementary Figure S13B and Figure 14) and H&E staining (Figure 6F and Supplementary Figure S13C and Figure 14) revealed typical features of damage response surrounding airways and alveoli where collagen was deposited in vehicle exposed mice. Moreover, immunohistochemical analysis of αSMA (Figure 6G and I), Vimentin and E-Cadherin (Supplementary Figure S13D, E) showed a fibrotic response after prolonged TGFβ1 treatment. This pathogenesis is markedly attenuated in mice receiving TH5487. Biochemical analyses of hydroxyproline content further confirmed that collagen disposition is reduced by TH5487 (Figure 6J). Together, these data suggest a critical OGG1 function in mediating tissue response to injury.

## DISCUSSION

**Figure 7.**
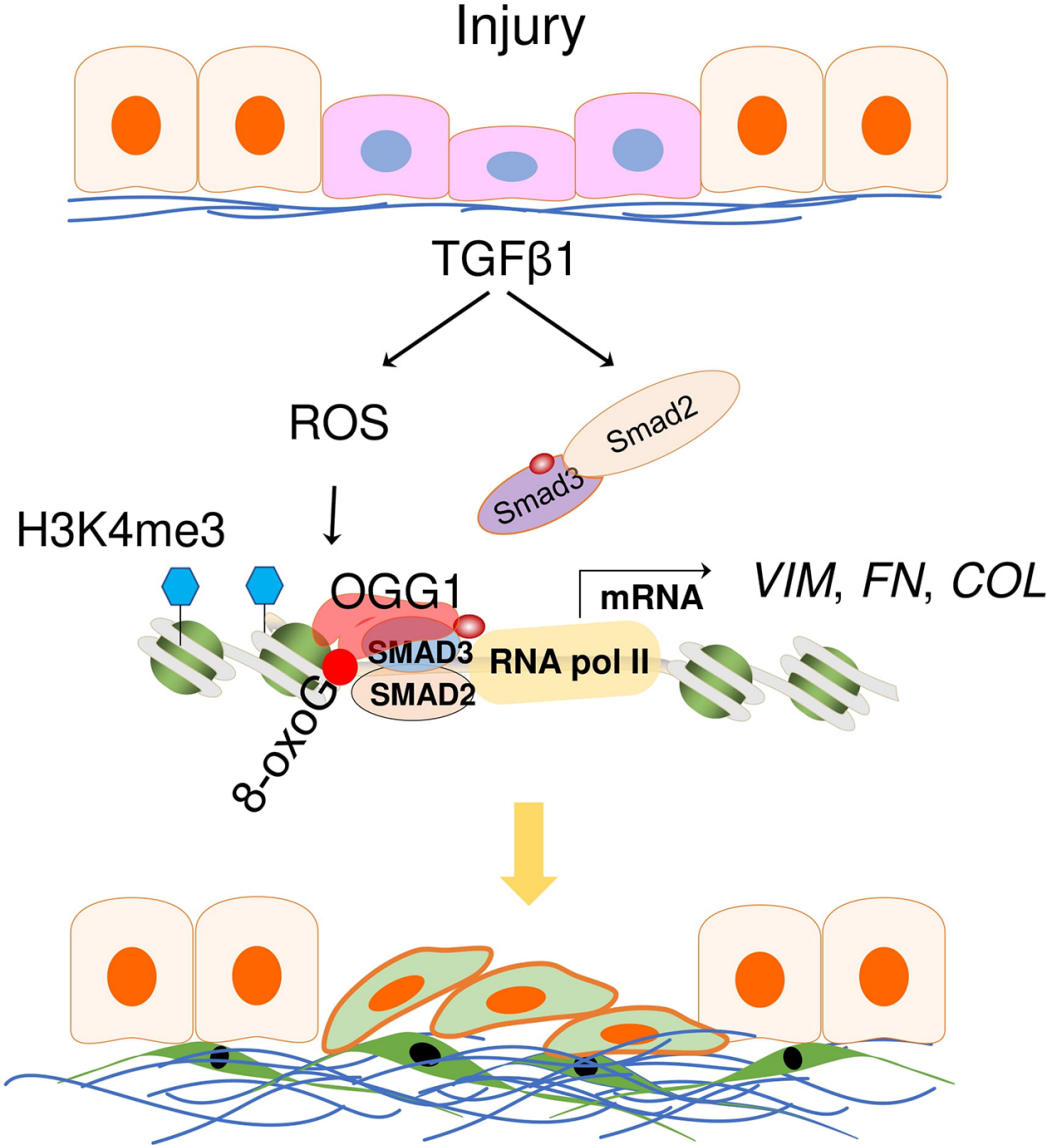
Proposed model of OGG1 in regulating fibrotic gene expression. Following airway epithelial injury, ROS and TGFβ1 signaling activate SMADs, and GC-rich promoters are oxidatively modified by ROS, leading to 8-oxoG specific binding by OGG1. OGG1 pre-excision function favors assembly of transcriptional machinery and ultimately activates fibrotic genes expression. Repeated injury/repair processes may result in tissue remodeling.

Effective tissue repair is essential in maintenance of homeostasis, a process intimately linking growth factors and matrix molecules. A prolonged fibrotic response and disrupted resolution occur when this process is dysregulated by impaired re-epithelialization and ECM expansion. Our biochemical and molecular studies have established a general model of transcriptional initiation by a BER protein, whereby OGG1 activates transcription through functional cooperativity via selected transcription factors (Figure 7). We propose that OGG1 is responsible for directing the binding of SMADs to key fibrogenic gene regulatory sites, and it is the sum of these interactions that determines oxidative stress responses to TGFβ1 signaling. These results, together with the mechanistic insights presented here, provide novel perspectives into transcriptional regulation via the OGG1-8-oxoG complex as an advancing regulator in tissue repair.

Our analysis of fibrotic gene promoters shows enrichment of 8-oxoG in response to TGFβ1 exposure, which highlights the importance of specific DNA damage actively responding to stressor through epigenetic reprogramming. These data are in line with an increase in 8-oxoG levels in DNA derived from cells treated with TGFβ1 as shown by UPLC–MS/MS (38). Non-random distribution of 8-oxoG was also demonstrated by selective capture methodologies and OxiDIP-sequencing to map 8-oxoG lesions in defined regions, including promoter and enhancer in mammalian cells (12,10,22,59), which shifted our view of 8-oxoG as a ROS generated epigenetic-like mark (60). During transcription, its excision is inhibited due to oxidation of cysteine residues in OGG1, while proceed after redox balance was restored. Also arguing for this hypothesis, studies from mutating cysteine residue of OGG1 have demonstrated that oxidant-induced modifications of OGG1 do not alter its DNA binding affinity to the 8-oxoG substrate but inhibit glycosylase activity (61,62). Another example of oxidizing conditions affecting OGG1 function is enhancing the interactions with transcription factors through OGG1 dimerization (63). Although we cannot rule out the possibility that other post-translational modifications could have effects on the OGG1 behavior, our results support the hypothesis that pre-catalytic function of OGG1 under oxidative stress plays a distinct role in the early transcriptional response. Accordingly, ChIP assays show undetectable level of APE1/Ref1 at the promoter during transcriptional initiation, which is confirmed by inhibitor studies providing additional evidence for lack of base excision and generation of AP-sites in early gene expression.

Our study reveals that 8-oxoG generation and OGG1 enrichment coincide with open chromatin markers H3K4me3, a feature associated with actively transcribed DNA (33,64). OGG1 searching for and recognizing 8-oxoG in the chromatin-based architecture are also required for transcription factors getting access to DNA and assembling transcription initiation complex, suggesting cooperative activities between DNA repair and transcriptional regulation (52). Consistent with previous study showing that timely orchestrated DNA oxidation associated with LSD1 is required for transcriptional initiation in tumor progression (38), we demonstrate that not only 8-oxoG and chromatin remodeler but also OGG1 are essential for EMT gene expression. Indeed, pharmacological inhibitions of LSD1 and BRD4 decrease OGG1 and SMAD3 binding to promoter regions. We show that OGG1 increases SMADs binding in a chromatin state, as SMADs DNA occupancy is significantly lower in the absence of OGG1. This indicates that cooperation of other factors is needed to increase promoter accessibility for SMAD-driven transcriptional activation. SMADs proteins have a relative low intrinsic DNA-binding activity (46). We show that OGG1 is a substrate-specific DNA binding partner that actively acts to recruit and stabilize their DNA binding complexes. Of mechanistic relevance, our EMSA analysis shows that OGG1 binding to 8-oxoG in proximity of SBE increases SMAD3 DNA accessibility. Crystal structure of the complex between OGG1 and 8-oxoG unveils features including base flipping, DNA kinking and duplex segmentation (65,66). Based on this evidence, it is tempting to speculate that the changes in DNA architecture induced by OGG1 lower the energy cost for accommodating SMAD3 DNA occupancy.

TGFβ1 is a pleiotropic cytokine with potent inflammatory activity (67). We observed that chronic challenging of mice with TGFβ1 results in fibrotic phenotypes associated with tissue remodeling and inflammation. Chronic epithelial injury activates the TGFβ1 expression to promote mucosal repair (68,69). In the lung, type-2 alveolar epithelial cells (AEC2s) can self-renew and differentiate into large, thin type-1 alveolar epithelial cells (AEC1s) to cover areas of damage or denudation after injury, allowing regeneration of alveolar epithelium to occur (70,71). Free radicals generated by TGFβ1 signaling or inflammatory cells result in genetic damage as evidenced by the presence of the oxidative DNA damage marker, 8-oxoG (72). Congruent with this work, we also observed elevated 8-oxoG levels in TGFβ1 exposed epithelial cells, and the pre-catalytic role of OGG1 is evolved co-transcriptionally when cells undergo EMT. These studies consistently imply 8-oxoG at physiological ROS levels is central in stress responses to support cellular adaptation, and thus provide a rational basis from which to design OGG1 substrate-specific interventions under chronic and acute pro-inflammatory environments. Treatment with TH5487 decreased fibrotic changes in lungs of mice, offering a perspective for a more refined redox medicine controlling specific ROS-mediated signaling pathways by selective targeting.

In conclusion, our findings exploring activated SMADs signaling, indicate that DNA damage response orchestrates ROS generation, master transcription factors and gene expression, with considerable implications for the understanding of cellular responses to environmental cues. This unique transcriptional reprogramming is associated with oxidative modification and enrichment for DNA repair pathways. These results strongly suggest that fine modulation of oxidative DNA damage and repair-mediated pathways are critical for tissue homeostasis related to transitional cell states and is a potential therapeutic target for treating lung diseases.

## DATA AVAILABILITY

The data underlying this article are available in the article and in its online supplementary material.

## Supplementary Material

gkac1241_Supplemental_File
