# Supplementary material for: 8-Oxoguanine targeted by 8-oxoguanine DNA glycosylase 1 (OGG1) is central to fibrogenic gene activation upon lung injury
Source: Nucleic Acids Res. 2023 Jan 18;51(3):1087–102. doi: 10.1093/nar/gkac1241 (PMC9943661; doi:10.1093/nar/gkac1241)
Supplement: gkac1241_Supplemental_File [file gkac1241_supplemental_file.pdf]

SUPPLEMENTAL MATERIAL

8-oxoguanine targeted by 8-oxoguanine DNA glycosylase 1 (OGG1) is central to fibrogenic gene activation upon lung injury

Lang Pan[1], Wenjing Hao[1,2], Yaoyao Xue[1], Ke Wang[1], Xu Zheng[1], Jixian Luo[1,3], Xueqing Ba[1,4], Yang Xiang[5], Xiaoqun Qin[5], Jesper Bergwik[6], Lloyd Tanner[6], Arne Egesten[6], Allan R. Brasier[7], Istvan Boldogh[1,✉]

[1] Department of Microbiology and Immunology, University of Texas Medical Branch, Galveston, Texas 77555, USA

[2] Institute of Genetics and Developmental Biology, Chinese Academy of Sciences, Beijing 100871, China

[3] School of Life Sciences, Shanxi University, Taiyuan, Shanxi 030006, China

[4] Key Laboratory of Molecular Epigenetics of Ministry of Education, School of Life Science, Northeast Normal University, Changchun, Jilin 130024, China

[5] Department of Physiology, School of Basic Medical Science, Central South University, Changsha, Hunan 410000, China

[6] Respiratory Medicine & Allergology, Department of Clinical Sciences Lund, Lund University and Skåne University Hospital, SE-221 84 Lund, Sweden.

[7]Department of Medicine, University of Wisconsin-Madison School of Medicine and Public Health (SMPH), Madison, WI, 53705, USA

✉ To whom correspondence should be addressed: Istvan Boldogh, Tel: +1(409)-772-9414; Fax: +1(409)-747-6869; Email: sboldogh@utmb.edu.

LIST OF CONTENTS:

Supplemental figures:

Supplementary Figure 1, related to Figure 1. Oxidative stress induced by TGFβ1 signaling.

Supplementary Figure 2: related to Figure 1. OGG1 is essential for the expression of fibrotic markers during EMT.

Supplementary Figure 3: related to Figure 2. The role of OGG1 loss of function in fibrotic gene expression.

Supplementary Figure 4: related to Figure 2. Expression level of *OGG1* mRNA is largely unchanged after TGFβ1 exposure.

Supplementary Figure 5: related to Figure 2. OGG1 binding to its substrates in DNA is essential for morphological changes during EMT.

Supplementary Figure 6: related to Figure 2. Effects of inhibitors on the expression of fibrotic genes.

Supplementary Figure 7: related to Figure 3. OGG1 exists in the same complex with pSMAD3 in nuclear compartment.

Supplementary Figure 8: related to Figure 4. OGG1 binding to 8-oxoG enhances SMAD3 recognizing SBEs.

Supplementary Figure 9: related to Figure 4. Loss of OGG1 function had minor effects on SMAD3 nuclear translocation and expression.

Supplementary Figure 10: related to Figure 4. Loss of OGG1 function had minor effects on NF-κB nuclear translocation and expression.

Supplementary Figure 11: related to Figure 5. Inhibition of OGG1 binding to its substrates in murine model decrease fibrotic gene expression.

Supplementary Figure 12: related to Figure 5. WB with full membrane for Figure 5F and 5H.

Supplementary Figure 13: related to Figure 6. Inhibit OGG1 binding to its substrates in murine model decrease tissue remodeling in lung.

Supplementary Figure 14: related to Figure 6. Whole lung scans from murine model following Masson Trichrome staining and H&E staining.

Supplemental Tables:

Supplementary Table 1: Lists of reagents used in this study.

Supplementary Table 2: Sequences of PCR primers for qRT-PCR

Supplementary Table 3: Sequences of PCR primers for ChIP coupled q-PCR

Supplementary Table 4: Sequences of siRNA (for silencing of indicated targets)

Supplementary Table 5. Gene expression in EMT pathway analyzed by plate-based quantitative PCR arrays (Mouse Epithelial to Mesenchymal Transition) using total lung RNA from individual mouse (n = 3).

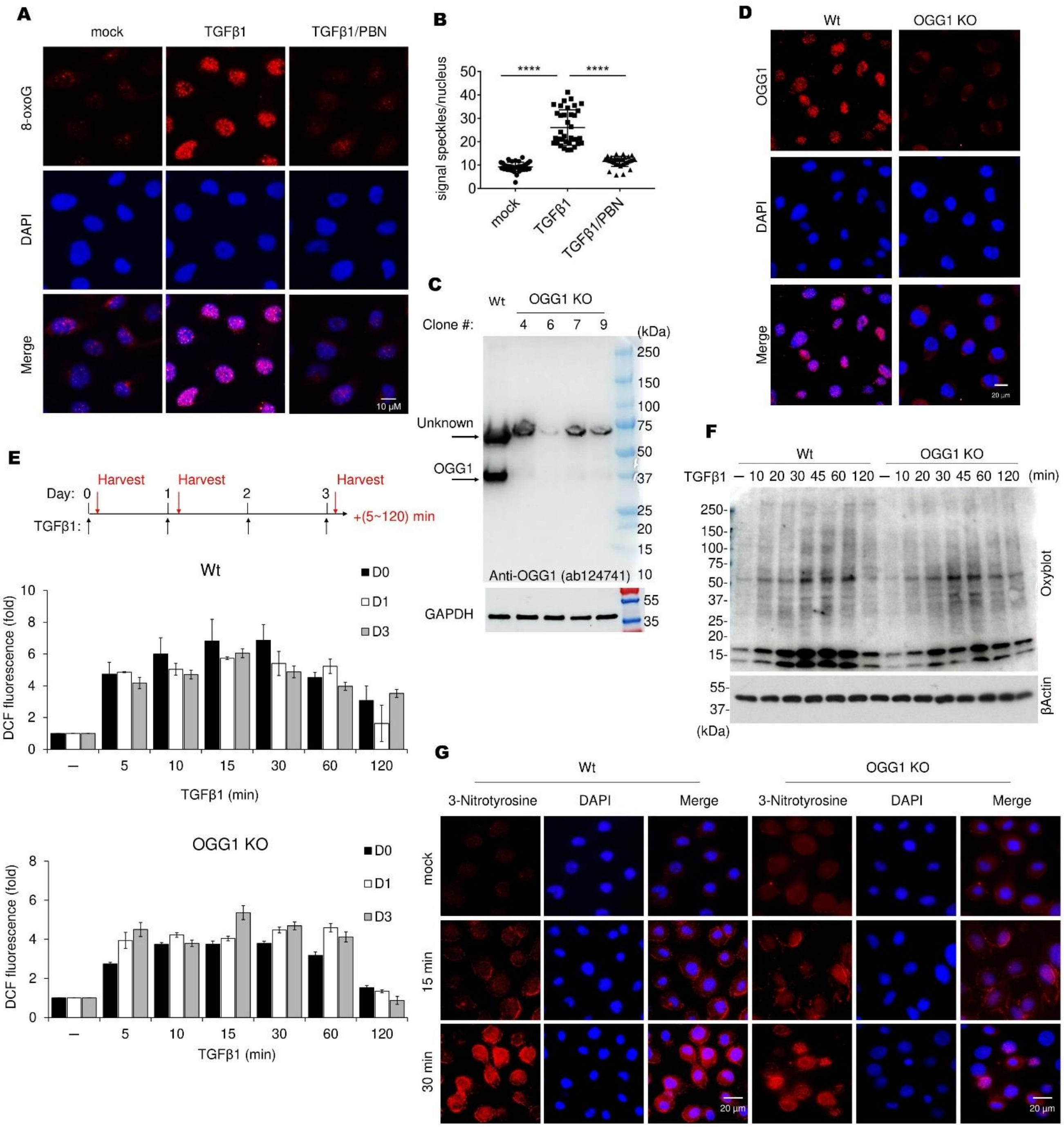

Supplementary Figure 1. Oxidative stress induced by TGFβ1 signaling. (A) Oxidative DNA damage as indicated by 8-oxoG staining in hSAECs upon TGFβ1 exposures ±PBN. (B) Quantification of signal speckles in nucleus. For each experimental condition, a minimum of 300 cells from a total of 3 independent were analyzed. (C) Immunoblots for OGG1 protein levels in Wt and OGG1 KO hSAECs. Clones #4-9 were tested and #6 was used in the following experiments. (D) Immunofluorescent staining using Ab against OGG1 in Wt and OGG1 KO cells. Scale bars, 20 μm. (E) Wt and OGG1 KO cells were exposed with TGFβ1 for indicated times, intracellular H_2O_2 production was evaluated by DCF-DA. The data presented are the mean of at least three experiments in duplicate

(n = 6). (F) Oxyblot analysis shows protein oxidation in Wt and OGG1 KO hSAECs following TGFβ1 treatment for indicated time. (G) Nitrosative stress as indicated by 3-nitrotyrosine staining in Wt and OGG1 KO cells ± TGFβ1 for indicated times (min). Representative images are shown from three independent experiments. Scale bars, 20 μm.

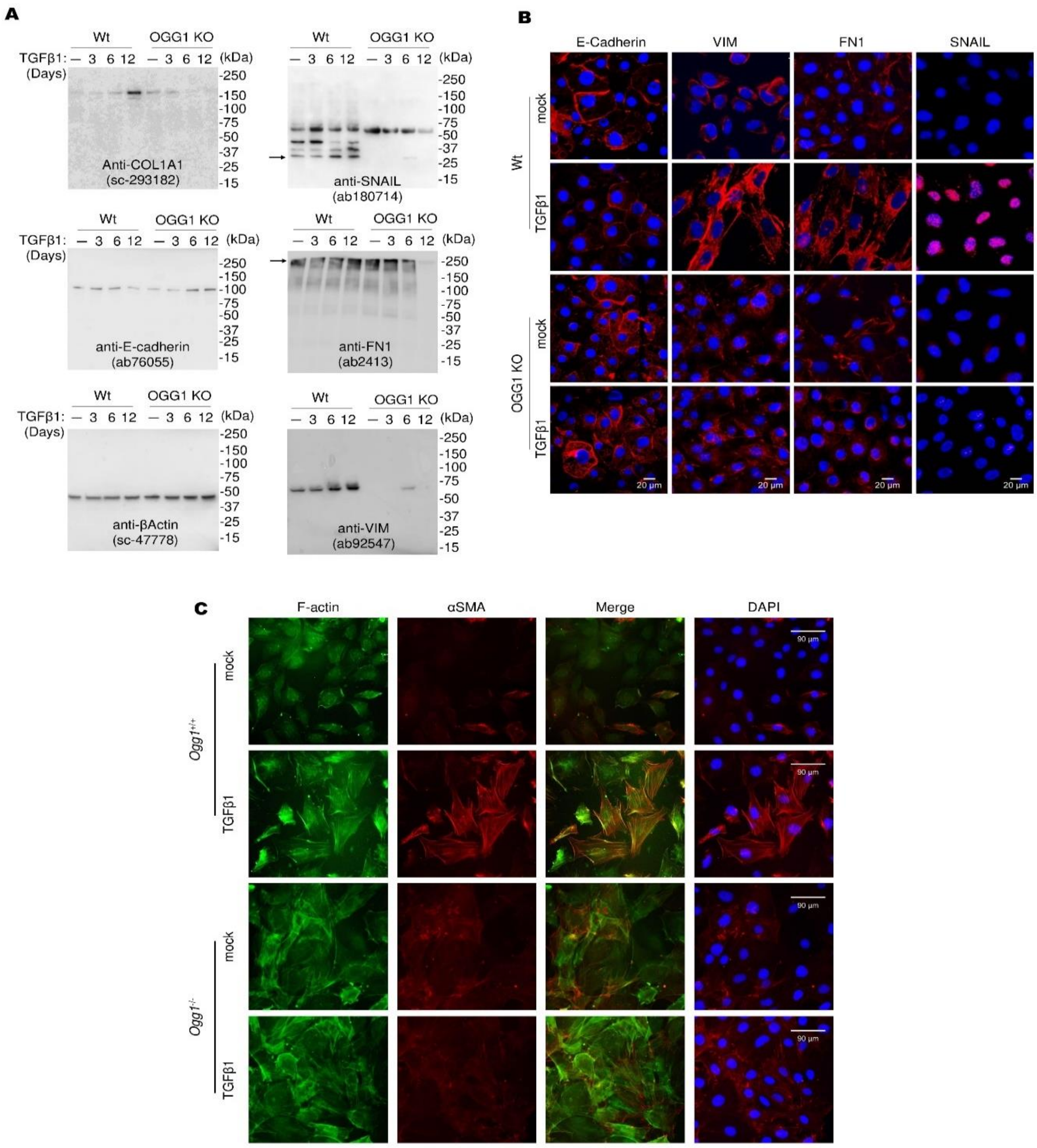

Supplementary Figure 2. OGG1 is essential for the expression of fibrotic markers during EMT. (A) Western blot validation of epithelial and mesenchymal markers in Wt and OGG1 KO hSAECs following TGFβ1 treatment for indicated days. βACTIN was used as a loading control. Representative images are shown from three independent experiments. (B) Representative images of IF showing expression and localization of epithelial and mesenchymal markers in Wt and OGG1 KO hSAECs following TGFβ1 treatment for 12 days. Representative images are shown from three independent experiments. Scale bars, 20 µm. (C) Ogg1+/+ and Ogg1-/- MEF cells were incubated with TGFβ1 for 4 days, and F-actin distribution and αSMA were stained with FITC-laved Phalloidin and Ab, respectively. Representative images are shown from three independent experiments. Scale bars, 90 µm.

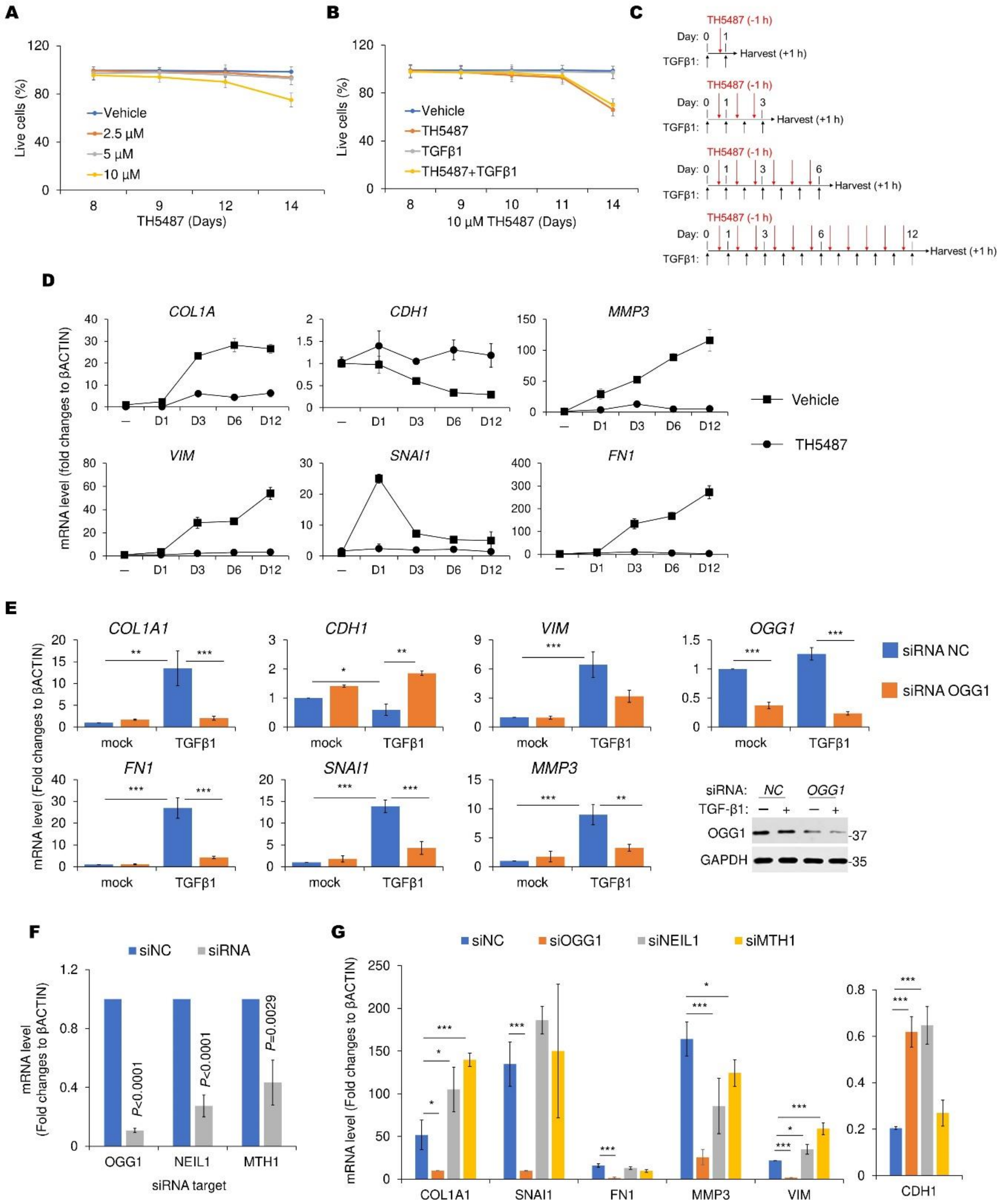

Supplementary Figure 3. The role of OGG1 loss of function in fibrotic gene expression. (A) hSAECs were incubated with indicated concentration of TH5487 for indicated days, and cell viability was evaluated by cytofluorimetry after PI staining. (B) hSAECs were incubated with TGFβ1 with or without TH5487 for indicated days, and cell viability was evaluated by cytofluorimetry after PI staining. (C) Schematic illustrating TGFβ1 induced EMT scheme and TH5487 treatment to analyze fibrotic gene expression. (D) TGFβ1 exposed hSAECs were treated with or without TH5487 for indicated days, and mRNA level was quantified with qRT-PCR analysis.

(E) Negative control (NC) or OGG1 targeted siRNA were transfected in hSAECs for 48 h and incubated with TGFβ1. Cells were harvested on day 3. mRNA level was quantified by qRT-PCR. WB showed protein level of OGG1 was downregulated by OGG1 targeted siRNA. (F) Negative control (NC), and OGG1, NEIL1 and MTH1 targeted siRNA were transfected in hSAECs. mRNA level was quantified with qRT-PCR analysis. (G) OGG1, NEIL1 and MTH1 targeted siRNA were transfected in hSAECs for 24 h and exposed with TGFβ1. Cells were harvested on day 3 and mRNA level was quantified with qRT-PCR analysis. *$P < 0.05$, **$P < 0.01$ and ***$P < 0.005$, by a two-tailed unpaired t-test.

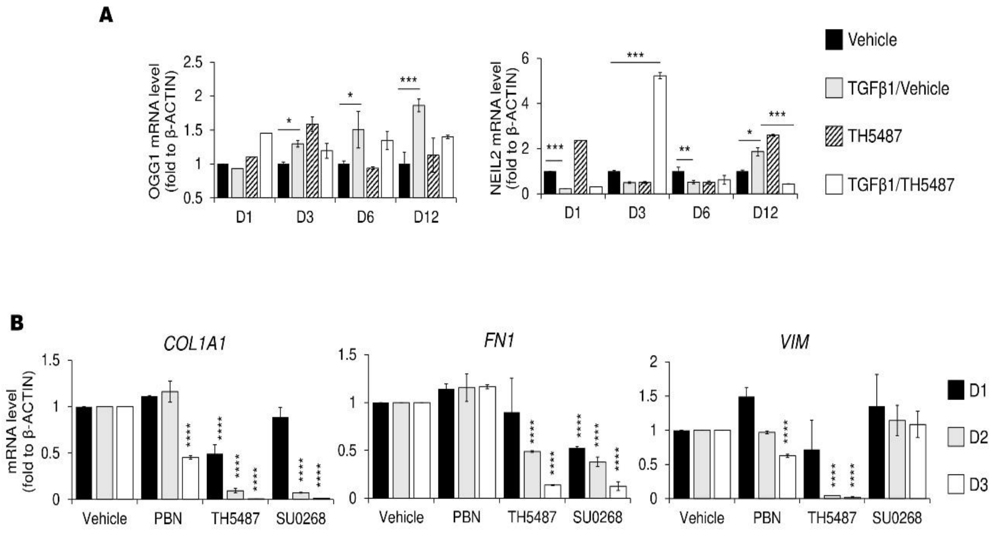

Supplementary Figure 4. Expression level of *OGG1* mRNA is largely unchanged after TGFβ1 exposure. (A) qRT-PCR analyses of *OGG1* and *NEIL2* mRNA levels in hSAECs. (B) MRC5 were incubated with PBN, TH5487 or SU0268 for indicated days, and qRT-PCR analysis was performed.

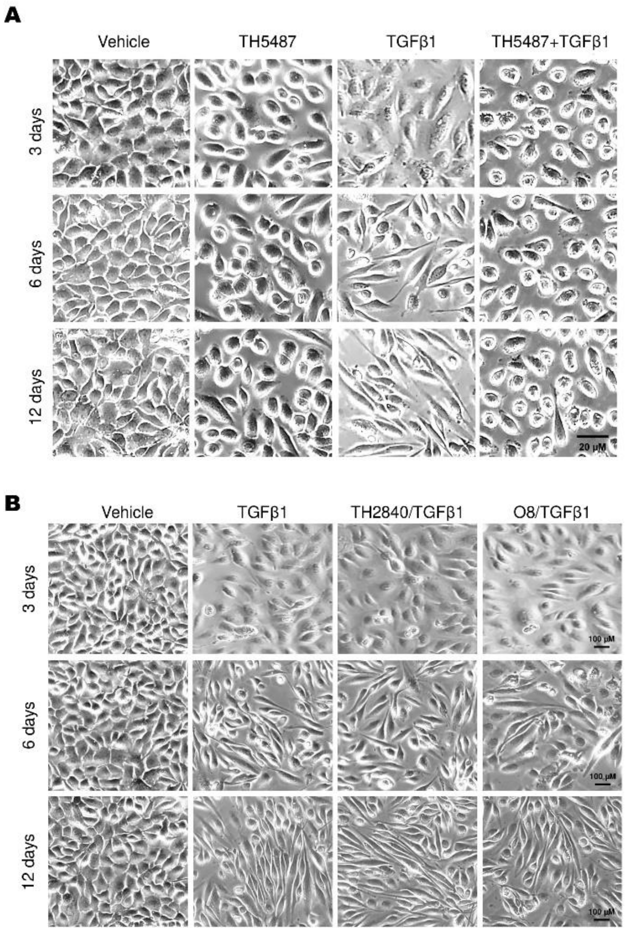

Supplementary Figure 5. OGG1 binding to its substrates in DNA is essential for morphological changes during EMT. (A) EMT was induced in hSAECs with TGFβ1 for indicated days, and preincubated with TH5487 did not induce spindle shape. Scale bars, 20 μm. (B) Inactive analogs TH2840 or inhibition of Schiff base formation during OGG1 catalysis through O8 treated cells showed spindle shape. Photographs of cells were taken with a phase/contrast microscope. Scale bars, 100 μm.

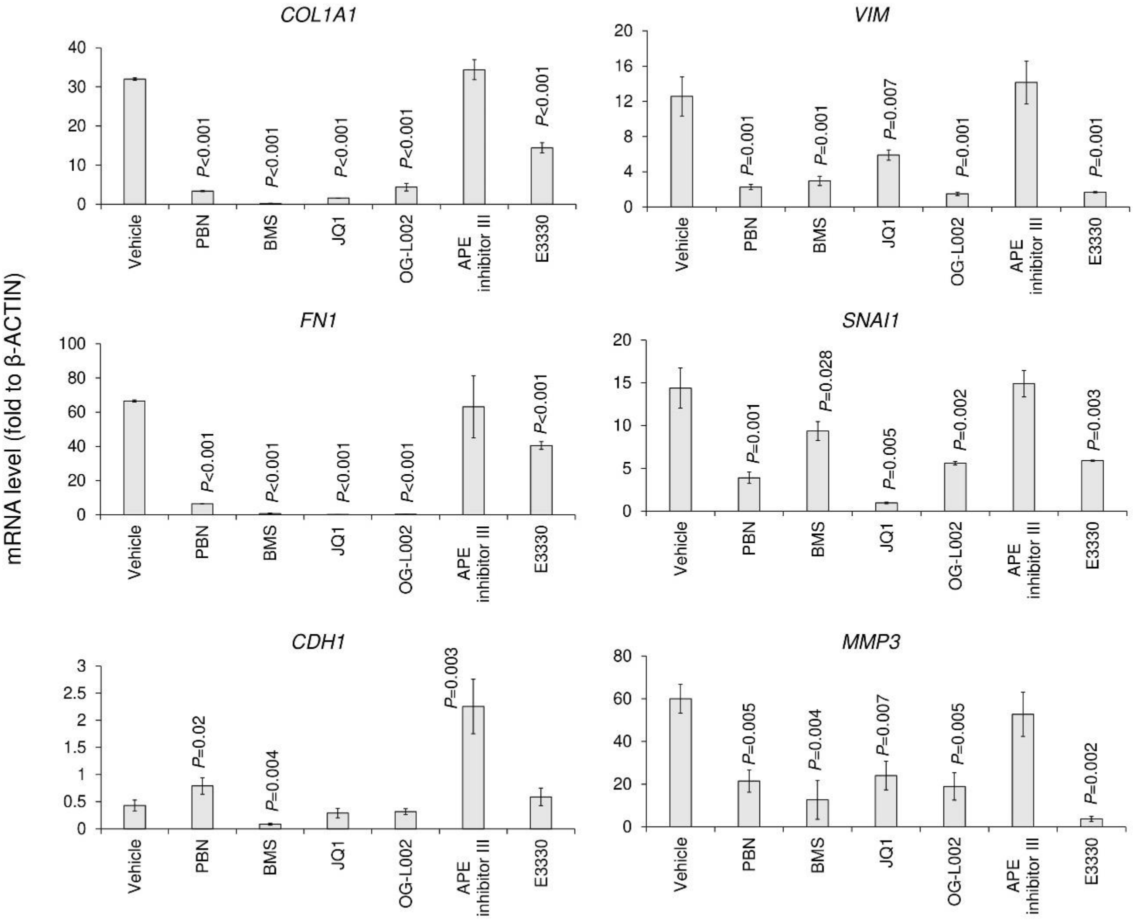

Supplementary Figure 6. Effects of inhibitors on the expression of fibrotic genes. hSAECs were pre-treated with Vehicle, PBN, BMS, JQ1, OG-L002, APE Inhibitor Ⅲ, or E3330 for 1 h, and exposed with TGFβ1 (as diagram shown in Supplementary Figure 3C). Fresh media was supplemented daily before challenge. Cells were harvested on day 3, and mRNA level was quantified by qRT-PCR. *$P < 0.05$, **$P < 0.01$ and ***$P < 0.005$, by a two-tailed unpaired t-test.

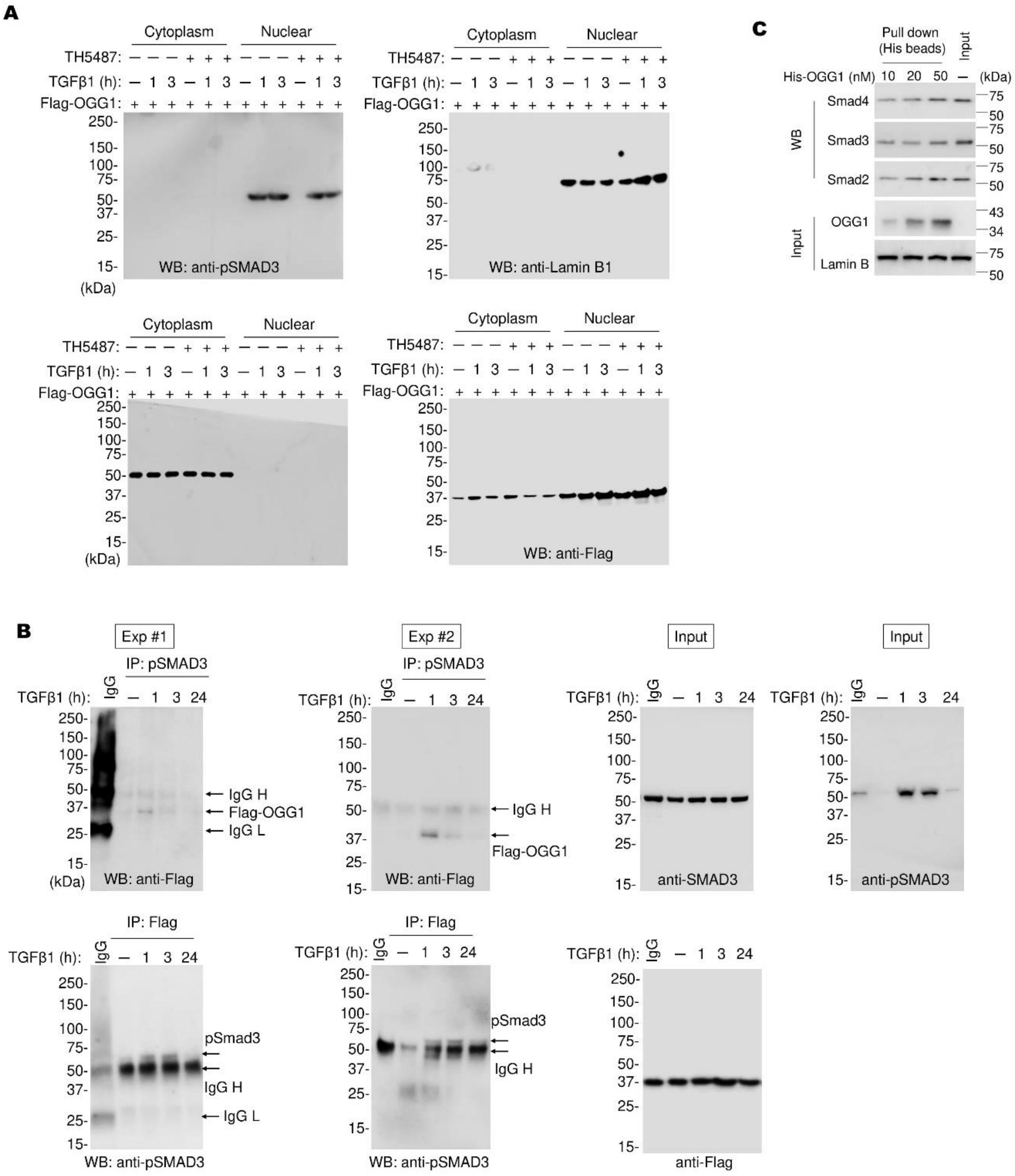

Supplementary Figure 7. OGG1 exists in the same complex with pSMAD3 in nuclear compartment. (A) Full membranes from Figure 3I. (B) hSAECs were transfected with Flag-OGG1 and nuclear protein is used in the co-IP experiment to test interaction between OGG1 and pSMAD3. Two representative images are shown from 3 independent experiments. (C) Increasing concentration of His-OGG1 was incubated with 2 µg of nuclear protein from TGFβ1-treated hSAECs for 1 h. Immunoblotting analysis of Smad2, Smad3 and Smad4 levels in His-Tag Dynabead based pulldown assays.

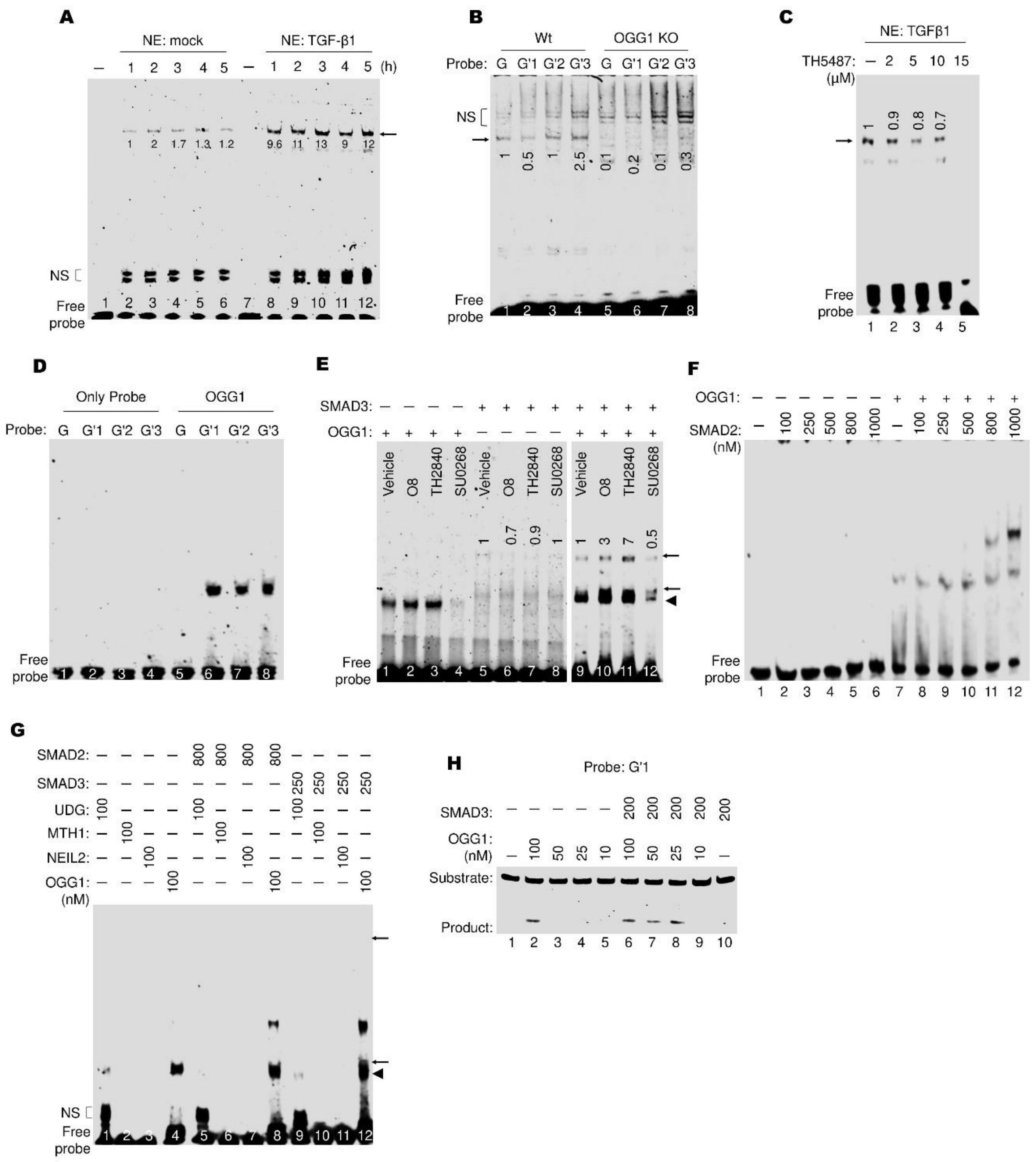

Supplementary Figure 8. OGG1 binding to 8-oxoG enhances SMAD3 recognizing SBEs. (A) G probe was incubated with nuclear extracts (NE) from mock or TGFβ1 exposed hSAECs for indicated times, and EMSA was performed to show activation of TGFβ1 signaling. (B) OGG1 deficiency abrogates SMAD-DNA shift. Wt and OGG1 KO cells were treated with TGFβ1 for 1 h, and NE was prepared to perform EMSA with G, G'1, G'2 and G'3 probes. (C) hSAECs were cultured with TGFβ1, and nuclear extracts were incubated with G'3 in the presence of increasing concentrations of TH5487. (D) Recombinant OGG1 binding to 8-oxoG containing oligo nucleotides (G'1-G'3) as shown in EMSA. (E) Inhibiting OGG1 binding to its DNA substrates with SU0268, but not inactive analog TH2840 or β-lyase activity inhibitor O8, decreases SMAD3 binding to SBE in G'3 probe. (F) Recombinant OGG1 and SMAD2 as indicated were incubated with G'3 probe and analyzed by EMSA. (G) OGG1 specifically

increases SMAD2 and SMAD3 binding to G'3 probe. UDG (100 nM), MTH1 (100 nM) and NEIL2 (100 nM) and OGG1 (100 nM) alone or incubated with SMAD2 (800 nM) or SMAD3 (250 nM) followed by the addition of G'3 probe prior to EMSA analysis. In C and E, arrows indicate migrating complexes containing SMAD dimer, triangle indicates OGG1-DNA complex. (H) SMAD3 increases OGG1 BER activity of 8-oxoG excision. G'1 probe was incubated with increasing concentrations of OGG1 at 37 °C for 60 min, and denaturing polyacrylamide gels showing separated substrates/products. Fold changes in intensity are labeled. Representative image shows gel shift from at least three independent experiments.

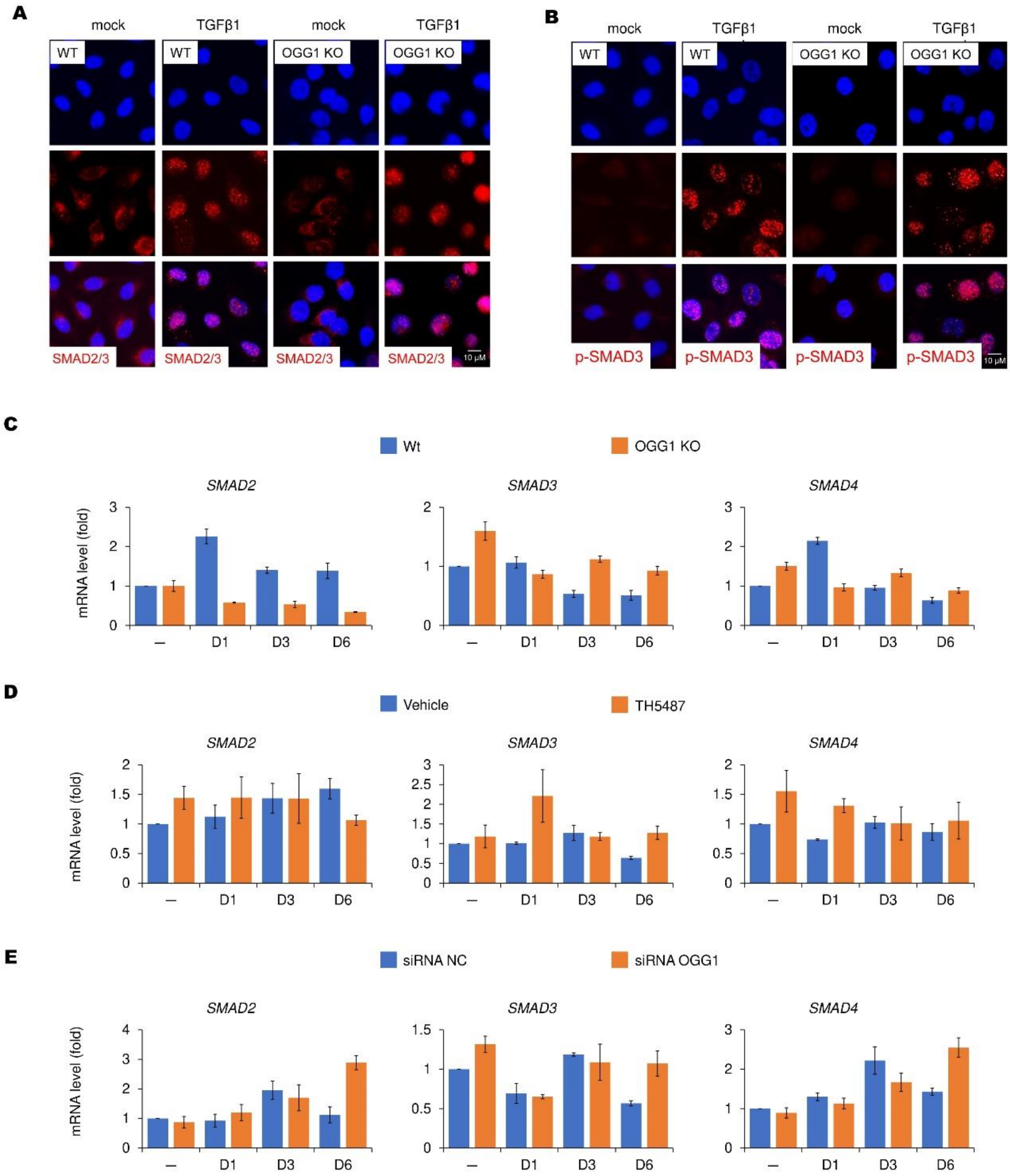

Supplementary Figure 9. Loss of OGG1 function had minor effects on SMAD3 nuclear translocation and expression. (A) IF staining of SMAD2/3 and (B) phosphorylated SMAD3 in Wt and OGG1 KO cells after TGFβ1 exposure for 1h. (C) OGG1 functional ablation by CRIPSR knock out, (D) small molecule of inhibiting OGG1 biding to its genomic substrates by TH5487, and (E) siRNA interference had minor effects on SMAD2, SMAD3 and SMAD4 mRNA levels during TGFβ1 treatment for indicated days.

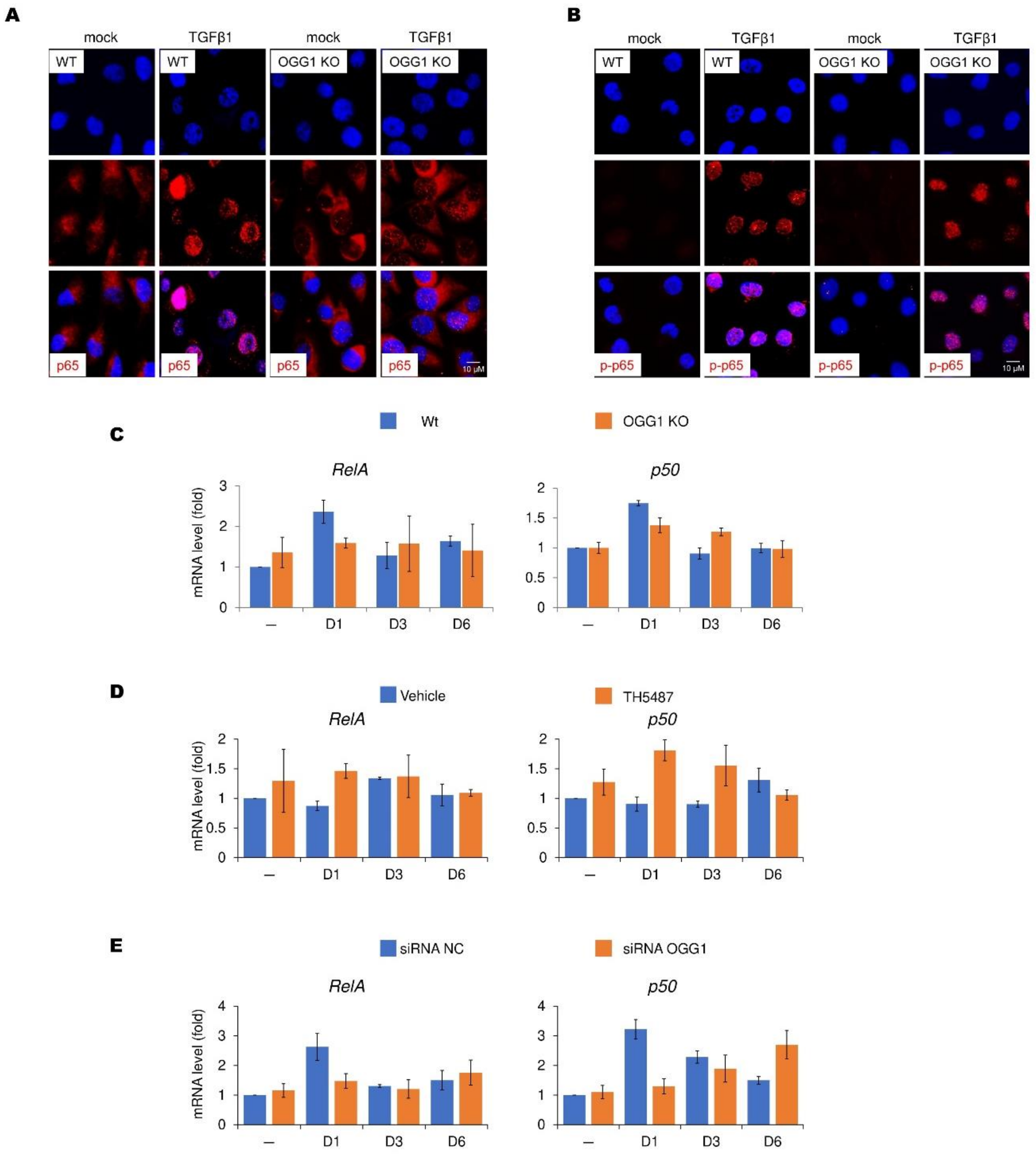

Supplementary Figure 10. Loss of OGG1 function had minor effects on NF-κB nuclear translocation and expression. (A) IF staining of p65 and (B) phosphorylated p65 in Wt and OGG1 KO cells after TGFβ1 exposure for 1 h. (C) OGG1 functional ablation by CRIPSR knock out, (D) small molecule of inhibiting OGG1 biding to its genomic substrates by TH5487, and (E) siRNA interference had minor effects on RelA and p50 mRNA level during TGFβ1 treatment for indicated days.

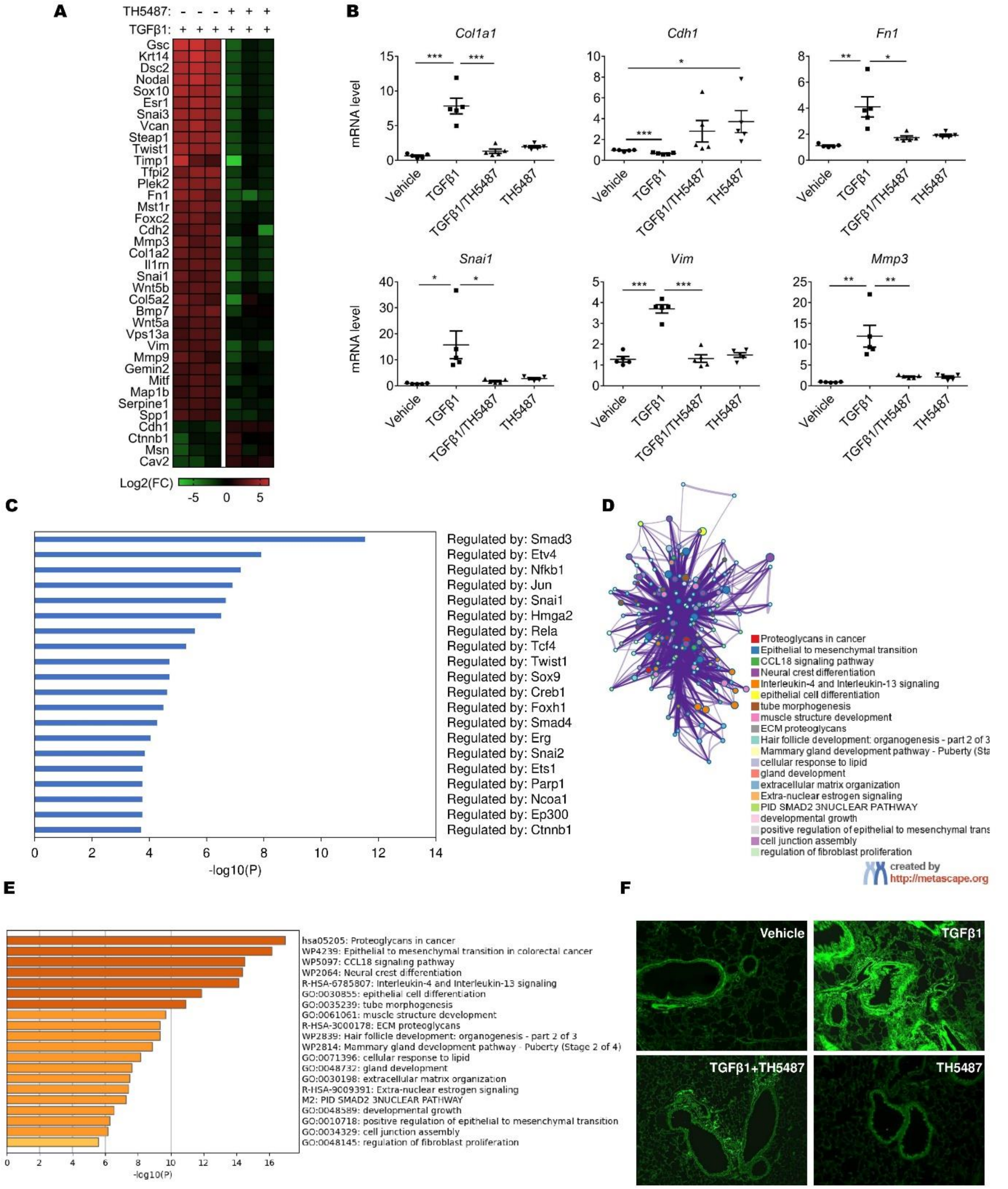

Supplementary Figure 11. Inhibition of OGG1 binding to its substrates in murine model decrease fibrotic gene expression. (A) Expression of OGG1 regulated genes from murine lungs as depicted in heat map. Data are represented as mean ± S.D from 3 individual mice. Scale indicates Log2 fold changes (FC). (B) qRT-qPCR analysis of epithelial and mesenchymal markers from murine lungs (normalized to βActin). Data are represented as mean ± S.D from 5 individual mice. **P* < 0.05, ***P* < 0.01 and ****P* < 0.005, by a two-tailed unpaired t-test. (C) Genes regulated by OGG1 from murine lung were analyzed in transcriptional regulatory relationships unraveled

by sentence-based text-mining (http://www.grnpedia.org/trrust; TRRUST). (D) Genes regulated by OGG1 from murine lung were analyzed in enriched network colored by cluster ID, where nodes that share the same cluster ID are typically close to each other. (E) Genes regulated by OGG1 from murine lung were analyzed with signaling pathways. Scale shows -log10 (P) obtained from Metascape (Zhou et al. Nature Commun. 2019 10(1):1523). (F) Collagen staining in murine lungs shows decreased deposition from TH5487 treatment after TGFβ1 exposure (n=5).

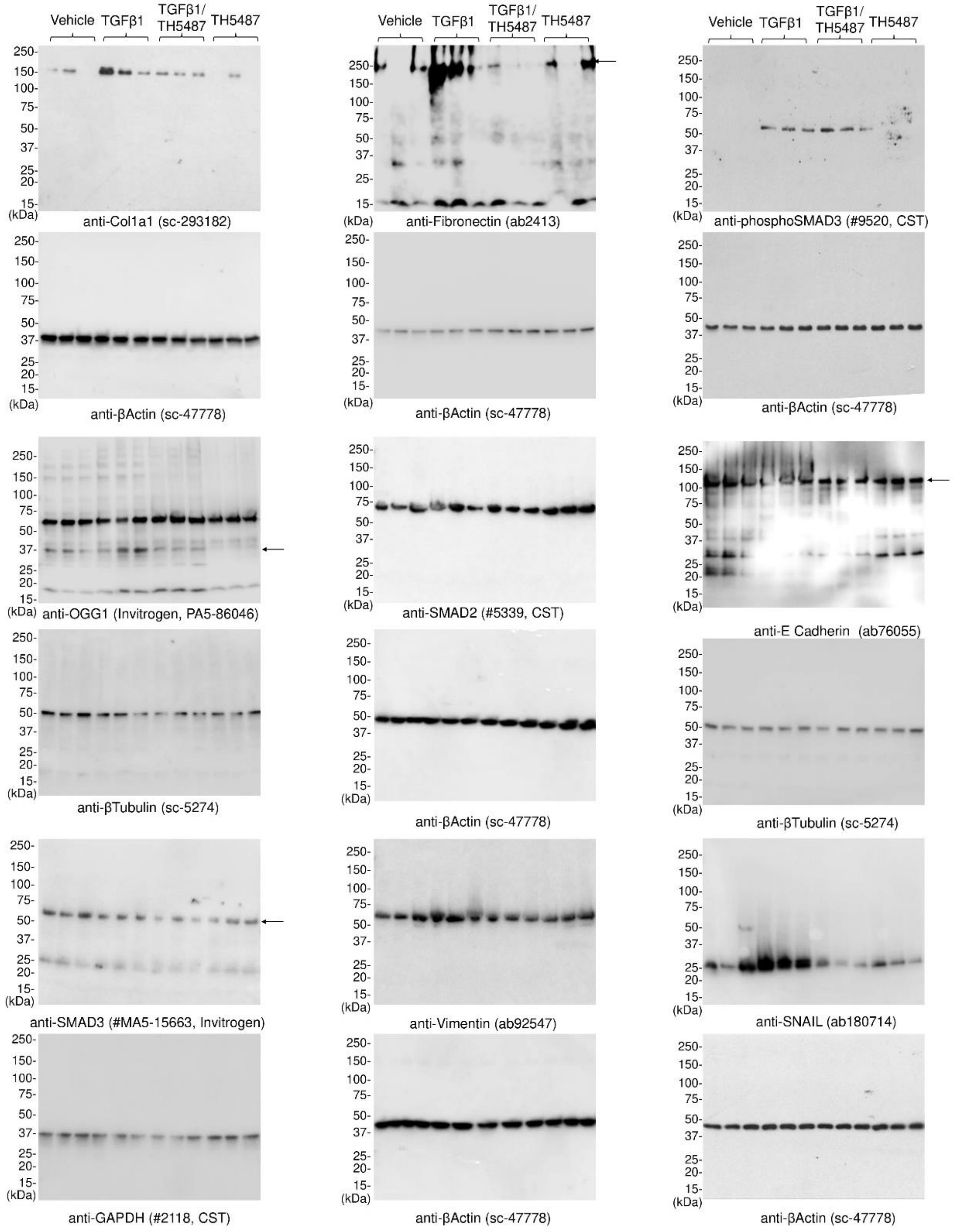

Supplementary Figure 12. WB with full membrane for Figure 5F and 5H.

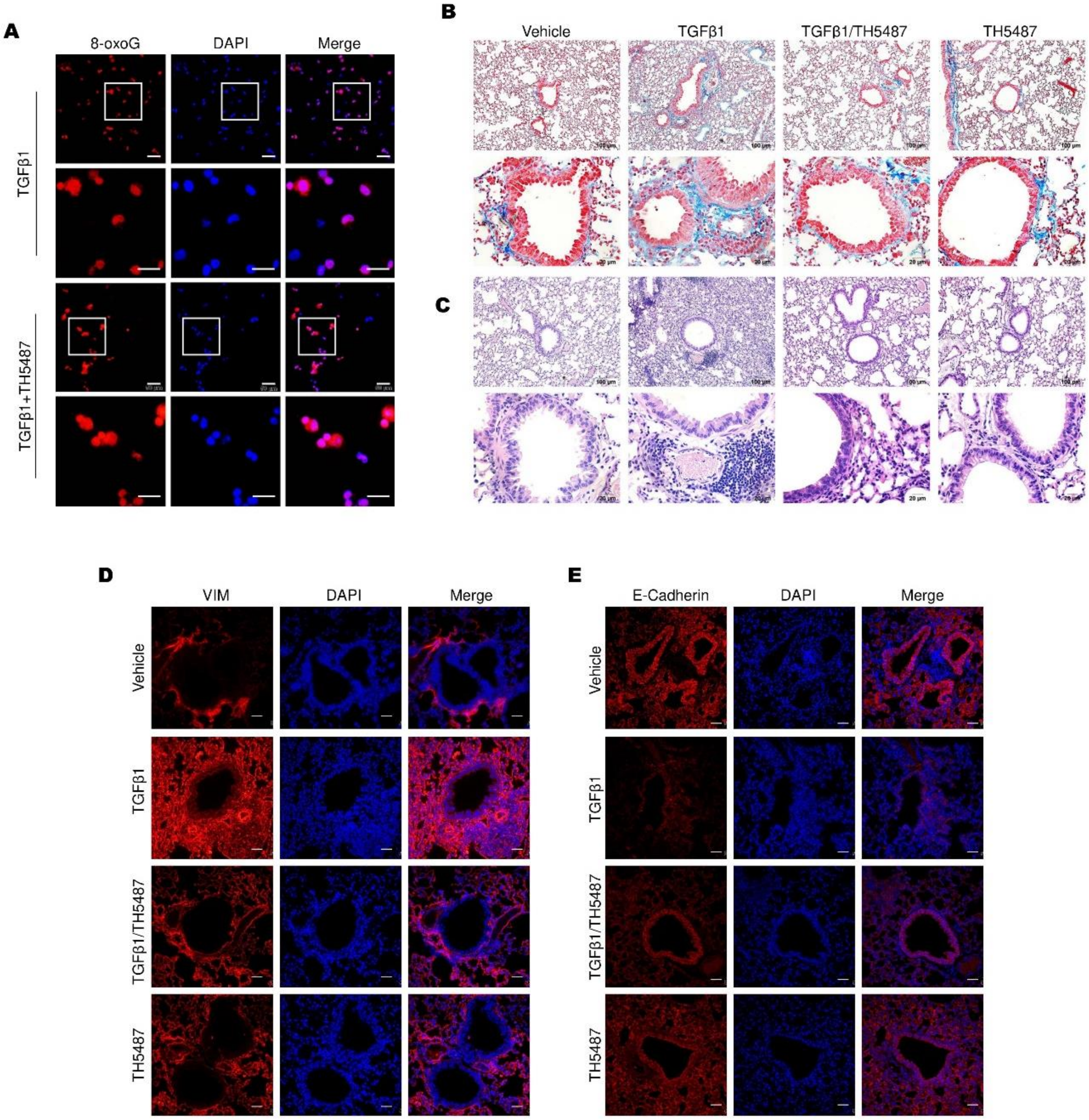

Supplementary Figure 13. Inhibit OGG1 binding to its substrates in murine model decrease tissue remodeling in lung. (A) Representative image shows immunofluorescent staining of 8-oxoG in murine BALF. Scale bars, 50 µm. (B) Representative image shows Masson Trichrome staining of murine model exposed to TGFβ1 treated with or without TH5487. (C) Representative image shows H&E staining of murine model exposed to TGFβ1 treated with or without TH5487. (D) Representative image shows staining of Vimentin in murine lung sections. Scale bars, 50 µm. (E) Representative images of staining E-cadherin in murine lung sections. Scale bars, 50 µm. In A-E, n=7 in each group.

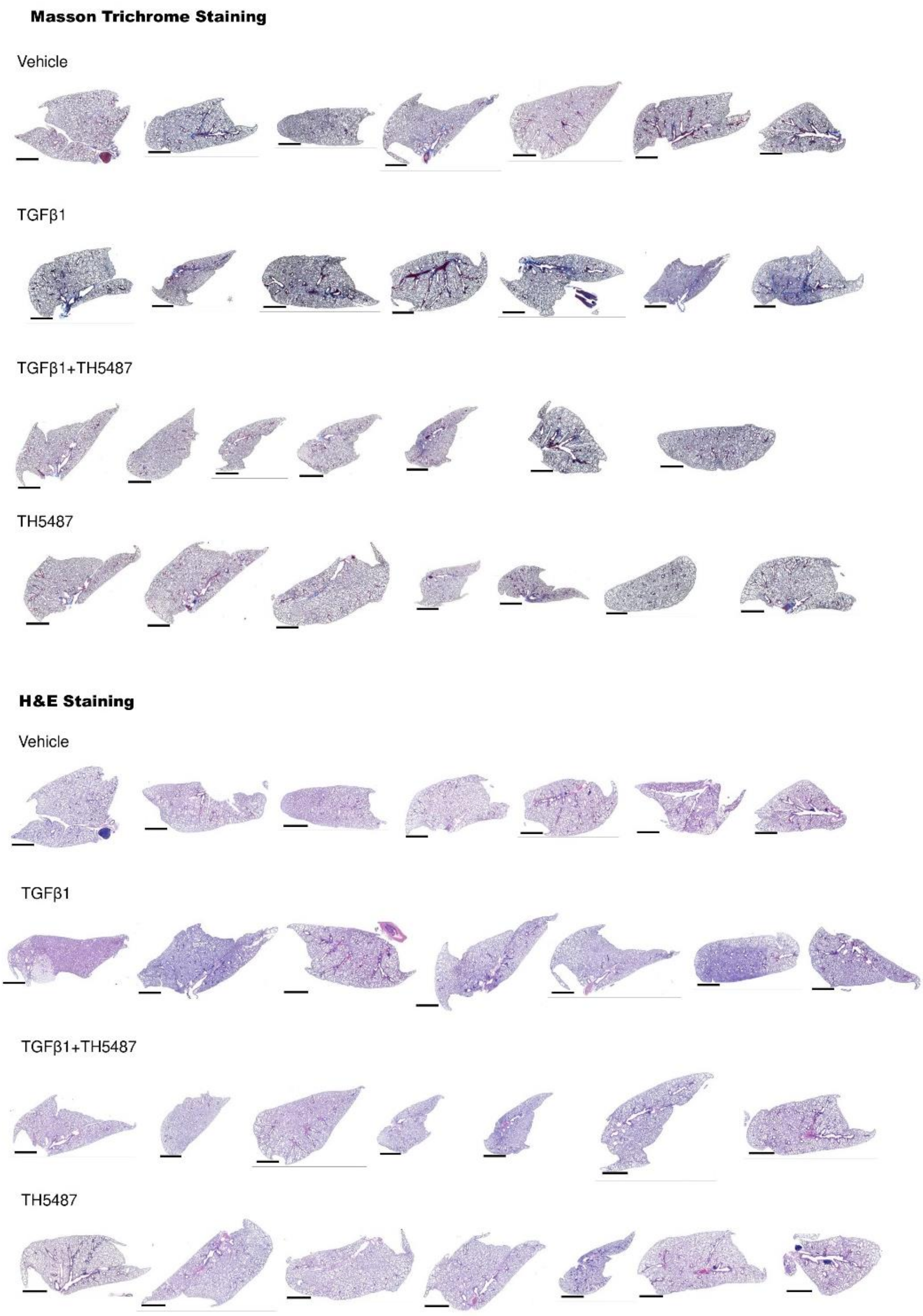

Supplementary Figure 14. Whole lung scans from murine model following Masson Trichrome staining and H&E staining (scale bar=2 mm).

Supplementary Table 1. List of antibodies and reagents used in this study

Recombinant protein for EMSA

His-OGG1	Novus Biologicals, NBP1-45318
GST-OGG1	antibodies-online Inc, ABIN1097188
His-SMAD3	Novus Biologicals, NBP1-30284
His-SMAD2	Novus Biologicals, NBC1-25856
His-SMAD4	Novus Biologicals, NBC1-22616
His-NEIL2	Novus Biologicals, NBP1-99001
His-MTH1	Novus Biologicals, NBP1-49309
His-UDG	Novus Biologicals, NBP2-75954

Reagents and kits:

OGG1 (for WB from human origin) antibody	Invitrogen, PA5-86046
OGG1 (for WB from human origin) antibody	Abcam, ab124741
8-OHdG antibody	Japan Institute for the Control of Aging, MOG020P
GAPDH antibody	Cell Signaling Technologies, #2118
Histone H1 (AE-4) antibody	Santa Cruz Biotechnology, sc-8030
αTubulin(B-7) antibody	Santa Cruz Biotechnology, sc-5286
Lamin A/C(636) antibody	Santa Cruz Biotechnology, sc-7292
Lamin B1 (A-11) antibody	Santa Cruz Biotechnology, sc-377000
FLAG antibody	Millipore-Sigma, F1804
Histone H3 (tri methyl K4) antibody	Abcam, ab8580
Histone H3 (tri methyl K9) antibody	Abcam, ab8898
Histone H3 antibody	Abcam, ab1791
SMAD2 (D43B4) antibody	Cell Signaling Technologies, #5339
SMAD3 (C67H9) antibody	Cell Signaling Technologies, #9523

SMAD4 (D3M6U) antibody	Cell Signaling Technologies, #38454
Phospho-SMAD3 (Ser423/425) (C25A9) antibody	Cell Signaling Technologies, #9520
control rabbit IgG	Santa Cruz Biotechnology, sc-2025
Vimentin antibody	Abcam, ab925470
E-Cadherin antibody	Abcam, ab76055
Fibronectin antibody	Abcam, ab2413
SNAIL antibody	Abcam, ab180714
alpha smooth muscle Actin antibody	Abcam, ab5694
Anti-rabbit Alexa 594	Thermo Fisher Scientific, A11012
Anti-mouse Alexa 488	Thermo Fisher Scientific, A11001
Benzonase® Nuclease	Millipore-Sigma, E1014
PhosSTOP Phosphatase Inhibitor Cocktail	Roche, 04 906 837 001
cOmplete Protease Inhibitors Cocktail	Roche, 04 906 837 001
Small Airway Epithelial Cell Growth Medium	PromoCell, C-21070
MEM (Minimum Essential Medium)	Thermo Fisher Scientific, 11095080
DMEM (Dulbecco's Modified Eagle's Medium)	Thermo Fisher Scientific, 11885084
FBS (fetal bovine serum)	Thermo Fisher Scientific, 10437028
Human TGF-β1	PERPOTECH, 100-21
N-tert-Butyl-α-phenylnitrone (PBN)	Millipore-Sigma, B7263
TH5487	Selleckchem, S8913
SU0268	Department of Pharmacology and Toxicology, UTMB

TH2840	Department of Pharmacology and Toxicology, UTMB
JQ1	Cayman Chemical, 11187
O8	Millipore-Sigma, SML1697
BMS345541	Cayman Chemical, 16667
OG-L002	AdooQ Bioscience, A13246
Apurinic Endonuclease 1 Inhibitor III (APE inhibitor III)	Millipore-Sigma, 262017
E3330	Novus Biologicals, NBP1-49581
Phalloidin (fluorescein isothiocyanate labeled)	Millipore-Sigma, P5282
Amplex Red	Invitrogen, A22188
5,6-carboxy-2', 7'-dichlorofluoresceindiacetate (DCHF-DA)	Millipore-Sigma, D6883
Deferoxamine mesylate (DFO)	Santa Cruz Biotechnology, sc-203331
10×RIPA	Millipore-Sigma, 20-188
Amersham Hybond-P PVDF Membrane	GE Healthcare, RPN303F
Pierce™ BCA Protein Assay Kit	Thermo Fisher Scientific, 23225
Propidium Iodide (PI)	Thermo Fisher Scientific, P1304MP
4',6'-diamidino-2-phenylindole dihydrochloride (DAPI)	Thermo Fisher Scientific, D1306
Duolink antifade medium	Millipore-Sigma, DUO82040
Duolink® In Situ PLA® kit	Millipore-Sigma, DUO92004
DNeasy Blood&Tissue kit	Qiagen, 69504
RNeasy Kits	Qiagen, 74106

iScript cDNA Synthesis Kit	Bio-Rad, 1708841
iTaq Universal SYBR green Supermix	Bio-Rad, 1725125
PCR arrays (Mouse Epithelial to Mesenchymal Transition)	QIAGEN, 330231 PAMM-090ZR
Protein A/G Mix Magnetic Beads	Millipore-Sigma, LSKMAGAG10
Dynabeads™ His-Tag	Invitrogen, 10104D
CelLytic™ NuCLEAR™ Extraction Kit	Millipore-Sigma, NXTRACT
IGEPAL® CA-630	Millipore-Sigma, I-3021
PMSF	Millipore-Sigma, P7626
ChIP-IT Express Magnetic Chromatin Immunoprecipitation kit	Active motif, 53008
Micrococcal Nuclease	New England BioLabs, M0247S
Phenol:Chloroform:Isoamyl Alcohol (25:24:1, v/v)	Thermo Fisher Scientific, 15593049
RNase A	Millipore-Sigma, R5500
Hydroxyproline Assay kit (Colorimetric)	Abcam, Ab222941
LipoFectamine 2000	Thermo Fisher Scientific, 11668-019
Oxidized Protein Western Blot Detection Kit	Abcam, ab178020
Wright-Giemsa Stain Pack	Thermo Fisher Scientific, 23-044626

Supplementary Table 2: Sequences (5’→3’) of PCR primers for qRT-PCR

**(h) COL1A1**<br>F: CCAGAAGAACTGGTACATCAGCA<br>R: CGCCATACTCGAACTGGAATC	**(m) Cdh1**<br>F: TGGGCCCTTTTATGGTGAGA<br>R: AGCATGCATAGTGGTCCCTT
**(h) FN1 E25**<br>F: TCAGAGCTCCTGCACTTTTG<br>R: GTAACGCACCAGGAAGTTG	**(m) Col1a1**<br>F: TTTGGAGAGAGCATGACCGA<br>R: GTAGGCTACGCTGTTCTTGC
**(h) FN1 E33**<br>F: CTCAGAATCCAAGCGGAGAG<br>R: CTGAACATTGGGTGGTGTCC	**(m) Mmp3**<br>F: AAGAGATCCAAGGAAGGCATC<br>R: ACATCCTTTGACAACTTGACGT
**(h) VIM**<br>F: GCTCAATGTTAAGATGGCCCTT<br>R: TGGAAGAGGCAGAGAAATCCTG	**(m) Snai1**<br>F: AGCCTCCTACCCCTCAGTAT<br>R: GGGGAGGGGAACTATTGCAT
**(h) CDH1**<br>F: CGAGAGCTACACGTTCACGG<br>R: GGGTGTCGAGGGAAAAATAGG	**(m) Vim**<br>F: CACGTCTTGACCTTGAACGG<br>R: AGTGAGGTCAGGCTTGGAAA
**(h) SNAI1**<br>F: GCGCTCTTTCCTCGTCAGG<br>R: GGGCTGCTGGAAGGTAAACTCT	**(m) Fn1**<br>F: AGTGTGATCCCCATGAAGCA<br>R: CAGGTCTACGGCAGTTGTCA
**(h) MMP3**<br>F: CCTGGAAATGTTTTGGCCCA<br>R: TCATCTTGAGACAGGCGGAA	**(m) Ogg1**<br>F: TGAGCTGCGTCTGGACTTGGTT<br>R: CTCCGTCTGAGTCAGTGTCCAT
**(h) RELA**<br>F: CTACGACCTGAATGCTGTGC<br>R: CTGCCAGAGTTTCGGTTCAC	**(m) Neil2**<br>F: GTGTGCTACACACTCTTGGACC<br>R: CGAGAGGAAGAACTCAGGCATG
**(h) NF-MADκB1(p50)**<br>F: GAGGATGGGATCTGCACTGT<br>R: TGCACCAAGAGTCCAGGATT	**(m) Neil1**<br>F: CTGCTGGAACTGTGTCACTTGG<br>R: AGCTCATGCCTGGCACACCATA
**(h) SMAD2**<br>F: AGTATGGACACAGGCTCTCC<br>R: GCTGTGATGCATGGAAGGTT	**(m) Mth1/Nudt1**<br>F: TGGACGTGCATATCTTCTCGGC<br>R: TCTGAAGCAGGAGTGGGAACCA
**(h) SMAD3**<br>F: GCAGAACGTCAACACCAAGT<br>R: CGAACTCACACAGCTCCATG	**(m) β-actin**<br>F: ATCTGGCACCACACCTTC<br>R: AGCCAGGTCCAGACGCA
**(h) SMAD4**<br>F: ACAAGTCAGCCTGCCAGTAT<br>R: GGTGCAGTCCTACTTCCAGT	
**(h) OGG1**<br>F: CATATGAGGAGGCCCACAAG<br>R: CAGAAGATAAGAGGACGCAGAAG	
**(h) β-ACTIN**<br>F: ACAGAGCCTCGCCTTTGCCG<br>R: ACATGCCGGAGCCGTTGTCG	

Supplementary Table 3: Sequences (5'→3') of PCR primers for ChIP coupled q-PCR

**(h) COL1A1 (-350/-176)**<br>F: TCCCCAGTTCCACTTCTTCT<br>R: CACATCTCCCCTCTTCGCA	**(m) Col1a1(-238/-13)**<br>F: CACCTCTGGCCCATGTAGAT<br>R: CCTGGGCCCCTTTTATACCA
**(h) FN1 (-587/-419)**<br>F: CAAAGAAAGGGAGCGGGATG<br>R: ATTGCGTCACCTCTCTTCG	**(m) Fn1(-343/-180)**<br>F: CTCCCTCCCTTTCCTTCGAG<br>R: CGCCCCGAACAAAAGAGATG
**(h) VIM (-324/-142)**<br>F: ACCGGACCCCTCTGGTTC<br>R: CCTCGAGCCTTCCTGCTC	**(m) Vim (-252/-98)**<br>F: GGGTGAGTAGAGAGTTCGGG'<br>R: GACCCGGAAAGAAGTGAGGA

Supplementary Table 4: Sequences of siRNA (for silencing of indicated targets)

**Human OGG1 siRNA-SMART pool (GeneID:4968)**<br>Target sequence1: 5'- CGACAAGACCCCAUCGAAU -3'<br>Target sequence2: 5'- GGACAAUCUUUCCGGUGGA -3'<br>Target sequence3: 5'- GCUCAGAAAUUCCAAGGUG -3'<br>Target sequence4: 5'- UACCCUGGCUCAACUGUAU -3'
**Human MTH1 siRNA-SMART pool (GeneID:4521)**<br>Target sequence1: 5'- GGGCAAAGUGCAAGAAGGA -3'<br>Target sequence2: 5'- GGAGAGCGGUCUGACAGUG -3'<br>Target sequence3: 5'- GAAAUUCCACGGGUACUUC -3'<br>Target sequence4: 5'- UGUUUGAGUUCGUGGGCGA -3'
**Human NEIL1 siRNA-SMART pool (GeneID:79661)**<br>Target sequence1: 5'- UACGAAACCUAGCGGAUAA -3'<br>Target sequence2: 5'- GACCAGAGGUUCUUCAAUG -3'<br>Target sequence3: 5'- UGACAUCCCAUCCUUGGAA -3'<br>Target sequence4: 5'- GGACCAAGCUGCAGAAUCC -3'
**Non-targeting siRNA**<br>5'- UGGUUUACAUGUCGACUAA -3'

Supplementary Table 5. Gene expression in EMT pathway analyzed by plate-based quantitative PCR arrays (Mouse Epithelial to Mesenchymal Transition) using total lung RNA from individual mouse (n = 3).

**Catalog #:**	PAMM-090Z			TGFβ1 vs. Control		TH5487/TGFβ1 vs. TGFβ1	
**NCBI Gene ID**	**RefSeq ID**	**Symbol**	**Description**	Z-score		Z-score	
12162	NM_007557	Bmp7	Bone morphogenetic protein 7	1.335489578	-0.264817141	-1.414078867	0.690136248
12390	NM_016900	Cav2	Caveolin 2	-0.723498617	-0.690587288	1.208241008	0.032366141
12550	NM_009864	Cdh1	Cadherin 1	-0.922058692	-0.467601811	1.255060298	-0.063067807
12558	NM_007664	Cdh2	Cadherin 2	0.148052382	1.143988739	-0.07547476	-1.185262077
12843	NM_007743	Col1a2	Collagen, type I, alpha 2	-0.634344286	1.411797876	-0.671516204	-0.742109928
12832	NM_007737	Col5a2	Collagen, type V, alpha 2	-0.145519317	1.291003516	-1.4137656	0.676058752
12387	NM_007614	Ctnnb1	Catenin (cadherin associated protein), beta 1	0.557526945	-1.404317904	1.41017836	-0.612635172
13506	NM_013505	Dsc2	Desmocollin 2	1.40717747	-0.581569575	-1.27891635	0.116696539
13982	NM_007956	Esr1	Estrogen receptor 1 (alpha)	1.297573323	-0.161726497	-1.259427618	0.07259483
14268	NM_010233	Fn1	Fibronectin 1	0.393057447	0.979961558	-0.178376112	-1.125775512
14234	NM_013519	Foxc2	Forkhead box C2	0.799257645	0.610762395	-1.310810524	0.195696845
14836	NM_010351	Gsc	Goosecoid homeobox	1.072789516	0.261629045	-1.30108648	0.170563575
16181	NM_031167	Il1rn	Interleukin 1 receptor antagonist	1.107754571	0.207476678	-1.409937002	0.60979373
16664	NM_016958	Krt14	Keratin 14	1.027926913	0.327181415	-1.2675154	0.090569395
17342	NM_008601	Mitf	Microphthalmia-associated transcription factor	1.412404295	-0.644270075	-1.404115088	0.555956156
17392	NM_010809	Mmp3	Matrix metallopeptidase 3	0.50046544	0.895258772	-1.191740045	-0.063536338
17395	NM_013599	Mmp9	Matrix metallopeptidase 9	0.085392524	1.179813897	-1.411522162	0.630236984
17698	NM_010833	Msn	Moesin	0.295498685	-1.345459823	1.345111168	-0.294392976
19882	NM_009074	Mst1r	Macrophage stimulating 1 receptor (c-met-related tyrosine kinase)	1.30623954	-0.183754562	-1.211616181	-0.025848202
17755	NM_008634	Map1b	Microtubule-associated protein 1B	1.2105069	0.027995935	0.003432469	-1.226457499
18119	NM_013611	Nodal	Nodal	1.311805668	-0.198328339	-1.391068609	0.474862391
27260	NM_013738	Plek2	Pleckstrin 2	-0.532222219	1.400815755	-1.251902937	0.056254814
18787	NM_008871	Serpine1	Serine (or cysteine) peptidase inhibitor, clade E, member 1	1.268256998	-0.092240005	-1.363173846	0.355522819
66603	NM_025656	Gemin2	Survival of motor neuron protein interacting protein 1	0.37663339	0.992196104	-1.19995474	-0.048159549
20613	NM_011427	Snai1	Snail homolog 1 (Drosophila)	-0.361136065	1.364707195	-1.089505482	-0.236101505
30927	NM_013914	Snai3	Snail homolog 3 (Drosophila)	0.701176574	0.713020456	-0.936934934	-0.448928138
20665	NM_011437	Sox10	SRY-box containing gene 10	0.47806372	0.913613493	-1.333957	0.260261101
20750	NM_009263	Spp1	Secreted phosphoprotein 1	-0.298295247	1.346338067	-0.69063654	-0.723450123
70358	NM_027399	Steap1	Six transmembrane epithelial antigen of the prostate 1	-0.622501927	1.410963887	-1.280600337	0.120639623
21789	NM_009364	Tfpi2	Tissue factor pathway inhibitor 2	0.036490872	1.206091655	-1.03487474	-0.317293548
21857	NM_011593	Timp1	Tissue inhibitor of metalloproteinase 1	0.541981239	0.860244216	-1.370775203	0.384170889
22160	NM_011658	Twist1	Twist homolog 1 (Drosophila)	1.045150621	0.302497495	-1.261084577	0.076241586
13003	NM_001081249	Vcan	Versican	1.369307533	-0.37847137	-1.262069938	0.078418825
22352	NM_011701	Vim	Vimentin	0.855054167	0.548004453	-0.969782433	-0.406535463
271564	NM_173028	Vps13a	Vacuolar protein sorting 13A (yeast)	1.055754714	0.287006983	-1.199220918	1.248765211
22418	NM_009524	Wnt5a	Wingless-related MMTV integration site 5A	1.115043654	0.195809599	0.404305872	-1.375780889
22419	NM_009525	Wnt5b	Wingless-related MMTV integration site 5B	1.364994885	-0.362198017	-1.079683217	-0.25117869

Catalog #:	PAMM-090Z			TGFβ1 vs. Control			TH5487/TGFβ1 vs. TGFβ1		
**NCBI Gene ID**	**RefSeq ID**	**Symbol**	**Description**	Fold Change			Fold Change		
12162	NM_007557	Bmp7	Bone morphogenetic protein 7	6.287534	6.332877	5.053458	-3.39937	-0.74622	-1.32835
12390	NM_016900	Cav2	Caveolin 2	5.461466	6.261001	5.238361	-2.76226	-0.90094	-1.46181
12550	NM_009864	Cdh1	Cadherin 1	5.41088	5.804745	4.949774	-2.45319	-0.78988	-1.29026
12558	NM_007664	Cdh2	Cadherin 2	5.32732	5.897258	4.7808	-2.84857	-0.63032	-1.32054
12843	NM_007743	Col1a2	Collagen, type I, alpha 2	5.257163	4.793707	4.675648	-3.26038	-0.79163	-1.2592
12832	NM_007737	Col5a2	Collagen, type V, alpha 2	4.523703	5.128299	4.4386	-2.35225	-0.61291	-1.16732
12387	NM_007614	Ctnnb1	Catenin (cadherin associated protein), beta 1	4.829263	4.826941	4.337507	-2.95292	-0.31189	-1.88224
13506	NM_013505	Dsc2	Desmocollin 2	4.477537	5.168106	3.984264	-2.32514	-0.35984	-0.95097
13982	NM_007956	Esr1	Estrogen receptor 1 (alpha)	4.616742	4.475549	4.463392	-2.60846	-0.47307	-1.04853
14268	NM_010233	Fn1	Fibronectin 1	5.008014	4.435542	4.080795	-2.9046	-0.74189	-1.36787
14234	NM_013519	Foxc2	Forkhead box C2	5.96054	2.871697	2.016011	-7.21076	-0.3768	-0.79823
14836	NM_010351	Gsc	Goosecoid homeobox	3.558584	4.386081	4.318644	-2.17719	-0.24737	-1.29377
16181	NM_031167	Il1rn	Interleukin 1 receptor antagonist	4.16528	3.96444	3.926446	-2.76953	-0.56603	-1.21996
16664	NM_016958	Krt14	Keratin 14	4.303567	4.047208	2.777836	-2.85674	-3.89793	-2.00519
17342	NM_008601	Mitf	Microphthalmia-associated transcription factor	3.278057	3.728406	3.469123	-2.19291	-0.66764	-1.25974
17392	NM_010809	Mmp3	Matrix metallopeptidase 3	3.431852	3.495859	2.470733	-1.54483	-0.41147	-0.74467
17395	NM_013599	Mmp9	Matrix metallopeptidase 9	2.66148	3.035322	3.246795	-0.8941	0.199084	-4.98658
17698	NM_010833	Msn	Moesin	3.583799	2.448613	2.906623	-3.22751	-0.61989	-1.48375
19882	NM_009074	Mst1r	Macrophage stimulating 1 receptor (c-met-related tyrosine kinase)	3.322159	2.825152	2.783044	-1.9124	-0.73874	-1.97508
17755	NM_008634	Map1b	Microtubule-associated protein 1B	2.78237	3.140681	2.862003	-2.45161	-0.65853	-1.33843
18119	NM_013611	Nodal	Nodal	3.235289	2.403385	2.680295	-3.83329	-1.1856	-2.31677
27260	NM_013738	Plek2	Pleckstrin 2	2.687067	2.877318	2.609598	-1.79975	-0.36327	-1.13385
18787	NM_008871	Serpine1	Serine (or cysteine) peptidase inhibitor, clade E, member 1	3.374954	2.698546	1.921654	-4.56352	0.903255	-0.24032
66603	NM_025656	Gemin2	Survival of motor neuron protein interacting protein 1	3.071789	2.844807	3.438153	-2.07708	0.208949	0.227259
20613	NM_011427	Snai1	Snail homolog 1 (Drosophila)	2.015511	2.668392	2.454276	-0.29712	-0.50602	-0.23645
30927	NM_013914	Snai3	Snail homolog 3 (Drosophila)	2.010863	2.580253	2.42099	-0.70957	-0.31239	-0.50938
20665	NM_011437	Sox10	SRY-box containing gene 10	2.340324	2.273944	2.350498	-2.98772	-0.87698	-1.91672
20750	NM_009263	Spp1	Secreted phosphoprotein 1	2.982874	2.481913	1.445879	-2.48439	-0.62605	-0.5514
70358	NM_027399	Steap1	Six transmembrane epithelial antigen of the prostate 1	2.062101	2.291057	2.363853	-1.1613	-0.00151	-0.50073
21789	NM_009364	Tfpi2	Tissue factor pathway inhibitor 2	1.959216	2.606377	2.004877	-1.83064	-0.54392	-0.65883
21857	NM_011593	Timp1	Tissue inhibitor of metalloproteinase 1	1.792678	2.464598	2.17868	-0.44633	-0.82219	-0.15069
22160	NM_011658	Twist1	Twist homolog 1 (Drosophila)	2.001939	2.211937	1.809595	-1.80701	-0.60697	-0.85052
13003	NM_001081249	Vcan	Versican	2.41094	1.922659	1.629982	-1.46729	-1.48646	-0.5821
22352	NM_011701	Vim	Vimentin	-1.31594	-0.78589	-1.48186	1.20097	1.090196	0.988053
271564	NM_173028	Vps13a	Vacuolar protein sorting 13A (yeast)	-2.83751	-0.59906	-0.7531	1.717812	-0.08185	0.190566
22418	NM_009524	Wnt5a	Wingless-related MMTV integration site 5A	-2.91778	-1.25233	-0.85746	1.704485	-1.03738	0.446002
22419	NM_009525	Wnt5b	Wingless-related MMTV integration site 5B	-2.18029	-2.22392	-0.64163	1.51151	0.736148	1.190237
